# Smartphone-Based Mindfulness and Mentalization Ecological Momentary Interventions for Common Mental Health Problems: Pilot Randomized Controlled Trial

**DOI:** 10.2196/79296

**Published:** 2025-09-30

**Authors:** Ciarán O'Driscoll, Sarah O'Reilly, Agata Julita Jaremba, Tobias Nolte, Madiha Shaikh

**Affiliations:** 1 Research Department of Clinical, Educational, and Health Psychology, University College London, Gower Street, London, WC1E 6BT, United Kingdom, 44 2076791991; 2 Anna Freud Centre, London, United Kingdom; 3 Clinical Health Psychology, North East London NHS Foundation Trust, Rainham, United Kingdom

**Keywords:** mindfulness, mentalization, ecological momentary intervention, just-in-time, expressed emotion, transdiagnostic

## Abstract

**Background:**

Accessible ecological momentary interventions deliver brief, real-time support integrated into daily routines. Interpersonal dynamics and maladaptive coping mechanisms can contribute to an individual’s anxiety and depression. Both mindfulness and mentalization represent psychological constructs with the potential to mitigate the negative impact of interpersonal stressors.

**Objective:**

This study aims to assess the feasibility and acceptability of an automated mindfulness- and mentalization-based ecological momentary intervention for common mental health problems as delivered via a mobile phone app.

**Methods:**

The design was a parallel-group pilot randomized controlled trial with 1:1 allocation ratio and exploratory framework. Recruitment of participants experiencing common mental health issues was internet-based from a university setting. Eligible participants were randomly allocated to fully automated mindfulness- or mentalization-based ecological momentary interventions via computer-generated randomization. Participants were blind to the alternative intervention options. Outcomes were self-assessed through questionnaires after 4 weeks. Primary outcomes were feasibility (recruitment, retention, and adherence) and acceptability (satisfaction ratings and qualitative feedback). Secondary outcomes included changes in depression (Patient Health Questionnaire-9 [PHQ-9]) and anxiety (Generalized Anxiety Disorder Questionnaire-7 [GAD-7]) scores.

**Results:**

A total of 84 participants were randomized (42 to each group). The interventions demonstrated good feasibility with an 89.2% retention rate and a mean adherence of 87.69% (SD 11.3%) across both groups. Acceptability ratings were positive, with favorable scores for ease of engagement (mean 5.20, SD 1.6), overall enjoyment (mean 5.15, SD 1.2), and likelihood of recommending the app (mean 5.11, SD 1.6) on a 7-point scale. For primary outcomes, both groups showed significant within-group reductions in PHQ-9 and GAD-7 scores, with moderate to large effect sizes (Cohen *d*=−0.68 to −0.81), with no significant difference between groups. Both treatments demonstrated clinically significant change, with 33 (44%) participants in both groups no longer meeting caseness criteria for anxiety and depression. Mindfulness performed better on improving assertiveness and perceived support compared to mentalization in the ecological momentary assessment data. One unintended harm was reported in the mindfulness arm, whereas none was reported in the mentalization arm.

**Conclusions:**

This pilot trial suggests that both mindfulness- and mentalization-based ecological momentary interventions are feasible and acceptable for individuals with common mental health problems and warrant further evaluation.

## Introduction

### Background

Common mental health problems, such as depression and anxiety, represent a significant global health concern due to their high prevalence and substantial impact on individuals and society [1]. Depression stands as a primary cause of global disability, while anxiety disorders add significantly to the worldwide disease burden [1]. Individuals experiencing these conditions endure emotional distress, functional impairment, and a diminished quality of life. Depression and anxiety generate extensive social and economic burden through diminished productivity, increased health care usage, and strain on social support systems [2]. Despite the availability of effective treatments, a considerable treatment gap exists, with a large proportion of individuals in need not receiving mental health care [3]. This highlights the urgent need for more accessible, scalable, and innovative interventions to address these prevalent conditions.

In response to this need, there has been increasing interest in the potential of microinterventions delivered through mobile health platforms. Microinterventions are characterized as brief, highly focused prompts designed to facilitate behavior change and promote well-being, delivered to individuals in their daily lives [4]. Microinterventions, by focusing on single, achievable goals, help the individual perceive the task as manageable and increase the willingness to engage [5]. This fosters habit formation, where the completion of small tasks generates a sense of achievement that increases motivation and promotes engagement, leading to sustainable health behaviors over time. Feedback plays a vital role in reducing the gap between current behavior and desired outcomes, where brief microinterventions brief microinterventions, when applied consistently, have shown the potential to positively influence psychological well-being through their impact on cognitive appraisals and emotion regulation [6]. Smartphones are an ideal platform for delivering microinterventions in a timely and convenient manner, integrating support seamlessly into individuals’ daily routines.

Ecological momentary interventions (EMIs) offer a promising approach to delivering targeted support in real time. This approach possesses unique benefits for managing mental health variability alongside environmental influences. EMIs have gained increasing recognition for their effectiveness in both treating and preventing depression and anxiety, largely due to advancements in mobile technology and sophisticated data analysis techniques [7]. EMIs can lead to a reduction in the severity of depression and anxiety symptoms, as well as improvements in related outcomes, such as stress and overall positive psychological functioning [7]. The adaptability of EMIs to fit personal requirements and contextual factors presents an opportunity to boost user interaction while alleviating the demands of conventional interventions [8]. Future advancements in technologies like digital sensors, combined with natural language processing and machine learning, will enhance EMIs to become more finely tuned and responsive to individual needs [9].

While symptom reduction is an important outcome in the treatment of depression and anxiety, interventions that target the underlying psychological processes contributing to these conditions may lead to more sustained and meaningful improvements [10]. Several key psychological factors are implicated in the development and maintenance of depression and anxiety, including negative self-concept, persistent rumination, difficulties in regulating emotions, and alterations in the processing of rewards [11]. Interpersonal dynamics and maladaptive coping mechanisms like avoidance can increase susceptibility to anxiety and depression [12]. Throughout developmental pathways, social determinants [13], such as relationship quality, adverse childhood experiences, family dynamics, cultural norms, and work environments, significantly impact mental well-being. Positive social interactions and strong social support networks can enhance motivation for healthy behaviors [14]. The absence of supportive networks combined with negative social interactions can contribute to the development and perpetuation of mental health problems [15].

Among these various social determinants, expressed emotion (EE) within key interpersonal relationships represents a particularly modifiable and well-researched target for intervention. EE is a concept in mental health research, referring to the attitudes and emotions expressed by family members or caregivers toward an individual with a mental illness [16]. High EE is characterized by a combination of critical comments, hostility, and emotional over-involvement. Individuals in high EE environments may express negative attitudes, intolerance, and a belief that the person with the mental illness has control over their condition and is choosing not to get better [17]. A substantial body of research has consistently linked high EE to higher rates of relapse and poorer outcomes across cultures and in a range of mental disorders [16], where the heightened stress associated with high EE family environments can exacerbate symptoms and impede the recovery process [18]. Individuals with anxiety and depression often exhibit a negative attention bias, characterized by a tendency to focus on and interpret information in a more negative light. This cognitive vulnerability can lead them to perceive criticism as true [19]. They may also be more reactive to perceived EE. For instance, a link between maternal EE and depression has been shown to be mediated by adolescents’ capacity for emotional regulation [20], and individuals with borderline personality disorder report higher negative affect and behavioral reactivity following cold and quarrelsome interpersonal interactions than healthy controls [21]. It is likely that the relationship between EE and psychiatric symptoms is bidirectional and involves vicious relational cycles between caregivers and patients [22]. For example, a depressive reactive coping style may elicit criticizing responses that negatively impact mood, leading to further unhelpful coping behaviors that maintain depression, such as cognitive or behavioral avoidance [23]. EE has also been an indirect target for family and caregiver interventions, with reductions in EE being associated with positive impacts on individuals with mental health difficulties and on family environments as a whole [24].

Mindfulness and mentalization represent 2 psychological constructs with the potential to mitigate the negative impact of perceived EE on individuals experiencing depression and anxiety. Mindfulness-based interventions cultivate a nonjudgmental awareness of one’s present moment experiences, including thoughts and feelings related to perceived criticism or hostility [25]. By fostering this awareness, mindfulness can promote emotional regulation, enabling individuals to observe their emotions without reacting impulsively. This capacity to become less reactive to unpleasant internal experiences is crucial for managing the emotional distress that can arise from perceived EE [26]. Mindfulness practices have been shown to reduce rumination and negative thought patterns, which are characteristic of depression and anxiety and can be triggered or intensified by perceptions of negative interpersonal interactions [27]. Mindfulness also overlaps with some cognitive and affective aspects of mentalizing [28] and may improve related aspects of social cognition, such as mental state attribution and empathetic concern [29] and support more accurate perceptions of others [24].

Mentalization-based interventions, on the other hand, focus on enhancing the ability to understand one’s own and others’ mental states, including the intentions and perspectives that may underlie behaviors perceived as critical or overinvolved [30]. Mentalizing capacity is reduced in depression [31] and has been shown to negatively predict well-being and emotional regulation in young adults [32]. Improved mentalizing skills may help individuals interpret EE in a less threatening or self-blaming manner, thereby reducing its detrimental effects on self-esteem and mood [33]. The capacity for mentalizing has been suggested as a potential protective factor against the negative impacts of high EE environments [34,35] and is associated with positive coping strategies in the face of stress [32]. Moreover, mentalization-based therapy aims to stabilize emotional expression and improve interpersonal functioning, which could be particularly beneficial in managing relationships where EE is a concern [30].

With these interventions, EE is not something that can be directly intervened on, as that would involve intervening with both the person expressing it and the person experiencing it; instead, this intervention can only address perceived EE. For instance, with mentalization microintervention, we are not trying to reduce EE itself; we are trying to strengthen mentalizing capacity, where better mentalizing acts as a buffer or protective factor. This reduces the impact of EE on depression, even though EE levels may be the same. These particular interventions do not remove the stressor but build capacity to handle it better.

The integration of mindfulness and mentalization microinterventions into smartphone-based EMIs presents a promising avenue for providing accessible and timely support to individuals with common mental health problems also experiencing interpersonal difficulties in their daily lives. Our approach is uniquely framed around EE, with interventions designed to be triggered in moments of perceived interpersonal stress related to EE (criticism, hostility, and overinvolvement). To our knowledge, this is the first study to design and test an EMI specifically based on mentalizing principles, which encourages understanding of and reflection upon mental states as a mechanism for improving interpersonal functioning and reducing distress. This targeted approach could complement other psychological interventions for individuals whose mental health is particularly affected by interpersonal dynamics.

### Study Objectives

The study objectives were as follows:

1. To determine the feasibility (eg, recruitment rates, adherence, and retention) and acceptability (eg, usability and satisfaction) of delivering mindfulness- and mentalization-based microinterventions via smartphone EMIs to individuals experiencing common mental health problems. ·
2. To investigate preliminary indications of efficacy by examining potential changes in relevant clinical outcomes.

## Methods

### Overview

Methods and results have been reported in compliance with the CONSORT-EHEALTH (Consolidated Standards of Reporting Trials of Electronic and Mobile Health Applications and Online Telehealth) guidelines [36]. The study was preregistered [37], and data and code are openly available [38].

### Trial Design

This pilot randomized controlled trial used a parallel-group design with a 1:1 allocation ratio. The trial was exploratory in nature, with the primary aim of assessing the feasibility and acceptability of the EMIs prior to a definitive trial.

The trial used both a between-groups and within-group (A/B) design. A randomized allocation sequence was generated using a computer program prior to starting recruitment, and participants were allocated based on the order in which they downloaded the app. COD generated the random allocation sequence, while SOR and AJ enrolled participants and assigned them to interventions. Participants were blinded to group allocation and to the theoretical basis of the intervention. Allocation was concealed through the app’s automated system. Researchers were not blind to group allocation due to practicalities of administering the study. Participants were screened (see below) before enrollment. Recruitment was internet-based, and intervention delivery took place via the m-path app [39].

### Participants

A total of 84 participants were recruited via convenience sampling through the university. Recruitment advertisements on an internet-based participant pool stated that the study was examining the impact of an app-based intervention to support people with anxiety or depression who feel that other people have an impact on their mental health.

Ecological momentary assessment (EMA) studies require commitment from participants over a long period (up to 4 times a day for 28 days). Therefore, dropout rates can be high. Appropriate incentives can encourage compliance. Therefore, we created a 2-fold incentive as outlined in the previous section. Participants were also incentivized through app functions, such as earning badges and data visualization.

### Eligibility Criteria

#### Inclusion Criteria

The participants were included if they (1) were aged 18 years or above; (2) were currently experiencing symptoms of anxiety or depression, or both (scoring in clinical range, a “caseness” threshold score of >9 on the Patient Health Questionnaire-9 [PHQ-9] [40], or >7 on the Generalized Anxiety Disorder Questionnaire-7 [GAD-7] [41]); (3) were able to access and use a personal smartphone; (4) had daily contact with a partner, family member, or friend; (5) were able to read and write in English; and (6) were registered with a general practitioner in the United Kingdom.

#### Exclusion Criteria

The participants were excluded if they were (1) currently receiving psychological therapy, (2) taking medication for their mental health, or (3) expressing any suicidal ideation or thoughts of self-harm at the point of screening (ie, scored >0 on question 9 of the PHQ-9).

### EMI Protocol

#### Screening

Participants accessed internet-based study information, consent forms, and screening questionnaires [42]. Screening assessed demographics, current mental health treatment, sleep patterns, general practitioner details, and baseline outcome measures.

#### Interventions

##### EMA Protocol

All participants downloaded the m-path app. Following this, they completed identical EMAs 4 times daily for 28 days, scheduled around individually reported sleep-wake times. EMAs were set at fixed intervals evenly spaced during the day. For each EMA, participants would receive an alert at the start of a 2-hour window and a reminder set after 60 mins. Each assessment measured mood, stress, perceived EE (criticism, hostility, and overinvolvement), warmth, and interpersonal behaviors. Participants received video training on app use. Engagement was monitored, with email reminders sent when completion rates fell below 80% (maximum once weekly).

##### EMI Phase

This phase began after participants completed 20 baseline EMA time points (typically 5 days) and then continued for the remainder of the 28-day study period. The EMI was activated when any EE item ratings (criticism, hostility, overinvolvement, or support) deviated >1 SD from their rolling average. Interventions were delivered immediately after the EMA and were delivered automatically via the mPath app.

##### Mentalization Arm

This microintervention was a structured reflection exercise on self and others’ mental states with a free-text response option to aid reflection. This was developed in line with the interpersonal affective focus, a repetitive pattern of relating to self and others [43]. The first step encouraged self-focused mentalizing, asking the client to reflect on their own perspective and understanding of a situation. Then other-focused mentalizing was encouraged, specifically asking the client to imagine the mental states (drives and motivations) and the intentions behind the other person’s actions. The final step encouraged self-focused mentalizing with a future orientation, asking the client to reflect on alternative responses and their own intentions for future behavior.

##### Mindfulness Arm

This microintervention used a 3-step mindfulness breathing exercise [44]. The first step encouraged internal awareness. The second step promoted present-moment awareness and acceptance of one’s current mental state, with an emphasis on physical sensations. The final step encouraged integration of multiple physical aspects of self-experience (whole body awareness). This could facilitate the connection between internal experience and how one might be perceived by others.

### Risk Management Protocol

Risk assessment occurred at screening (question 9 of the PHQ-9) and study completion. In terms of structured risk management, Skills Training On Risk Management (STORM) [45] was implemented for participants reporting suicidal ideation, including signposting or telephone assessment by the research team.

### Protocol Changes

The following modifications were made to the protocol following preregistration but prior to the pilot trial:

1. Aims: The aims shifted from a more mechanistic study focused on EE to a broader feasibility study with exploratory efficacy outcomes. Mindfulness was framed as an active control in the preregistration. All preregistered aims were addressed within the study, though with different emphasis than originally planned.
2. Intervention adjustments: Following pilot testing with 3 volunteers prior to participant recruitment, an initial prompt was incorporated into the mentalization-based intervention to facilitate self-mentalizing. This modification was made in response to feedback indicating that participants found it difficult to engage in mentalizing others’ perspectives without first reflecting on their own internal experiences.
3. Participation incentives: The minimum participation rate required for payment was lowered from 80% to 60% to increase feedback capture via the postintervention questionnaire.

The development and content were finalized and remained unchanged throughout the trial.

### Data Collection

#### Descriptive Variables

Demographic data regarding sex, age, ethnicity, and employment status were requested in the screening questionnaire. Maladaptive personality traits were measured using the Personality Inventory for DSM-5 Brief Form (PID-5-BF) [46], a 25-item self-report measure that aligns with the diagnostic criteria for personality disorders and assesses 5 personality trait domains, including negative affect, detachment, antagonism, disinhibition, and psychoticism. Internal consistency for the total score within the sample was a Cronbach α of 0.86.

#### Feasibility Outcomes

We assessed feasibility through the following outcomes:

1. Recruitment rate was evaluated by tracking the number of individuals screened, the number meeting eligibility criteria, and the percentage of eligible participants who subsequently consented and were randomized to a study arm.
2. Retention rates were defined as the percentage of randomized participants who completed the final study assessment at the end of intervention. Data were systematically collected on the number of participants at each stage of the study pipeline, from initial screening through to final data collection.
3. Adherence rates were operationalized as the percentage of participants who engaged with at least 80% of the EMAs over the 4-week intervention period. The 80% cutoff was based on empirical standards in this area [47].
4. Engagement with the intervention content was measured by the average number of mindfulness or mentalizing exercises completed per participant throughout the intervention period, as recorded by the app. These metrics allowed us to gauge participants' sustained interaction with both the assessment components and the therapeutic elements of the EMI.

#### Acceptability Outcomes

We assessed acceptability postintervention through a dedicated postintervention questionnaire. This assessment aimed to understand participants’ perceptions of the intervention’s usability, utility, and overall experience. In addition to quantitative ratings, participants were provided with open-ended questions to offer qualitative feedback on their overall experience, including perceived benefits, challenges, suggestions for improvement, and any other relevant comments regarding the intervention and the EMI delivery method.

#### Potential Effectiveness Outcomes

##### Primary Outcomes

We collected the following preliminary outcome data for a future definitive trial.

###### Common Mental Health Problems

These problems were assessed pre- and postintervention using the PHQ-9 to assess depression [40] and the GAD-7 to assess generalized anxiety [41]. Internal consistency within the sample was α=0.72 for each measure.

###### EMA Measures

Participants completed EMAs throughout the day via smartphone notifications. Each assessment consisted of 10 single-item measures designed to capture momentary interpersonal experiences, emotional states, and behavioral responses. All items were rated on a scale of 0-10, with higher scores indicating greater intensity of the assessed construct. The prompt for each item began with “Since the last beep...” to capture experiences between assessment points. Items assessed interpersonal experiences (criticism, hostility, overinvolvement, support, and warmth), emotional states (mood and stress), behavioral responses (engagement), and interpersonal stance (interaction and assertiveness). Specifically, participants rated their agreement with statements such as “Since the last beep I have felt criticized” (criticism) and “I feel valued or close to others” (support). For mood, participants responded to “How do you rate your mood generally?” with higher scores indicating more positive mood. For interaction, participants rated their reactions along a continuum from “Cold or Hostile” to “Warm or Friendly.” A full list of EMA items and the interventions can be found in the Open Science Framework (OSF) storage [38].

### Secondary Outcomes

Perceived EE was measured using the Level of Expressed Emotion (LEE) [48], a self-report measure indicating the perceived emotional environment in an individual’s close relationships. Participants choose one person who was most influential in their life during the past 3 months and indicate whether said person displayed any of the behaviors indicated in the items. Internal consistency within the sample was α=0.95.

Mentalizing capacity was measured using the Reflective Functioning Questionnaire-8 (RFQ-8) [49]. Internal consistency within the sample was α=0.79.

### Sample Size

We determined a sample size of 80 participants (40 in each group) based on methodological considerations for feasibility studies involving EMA [50]. The sample size calculation considered the practical constraints of managing intensive data collection (approximately 4 assessments per day for 28 days, yielding 112 potential observations per participant) while ensuring adequate representation to estimate parameters for the main study. With an anticipated 70% completion rate of EMA prompts, we expected approximately 6272 completed assessments across all participants, providing sufficient statistical power to conduct preliminary analyses of temporal patterns and associations between measured variables. Our sample size provided robust data to estimate between-group differences and within-person variability [51]. This sample size was also sufficient to estimate compliance rates, adherence patterns, and variability in EMA responses; to test the usability of the EMA protocol; and to detect technical issues that might affect implementation in a larger trial [52,53].

### Statistical Methods

Baseline differences between intervention groups on demographic, personality, and outcome variables were assessed via Student independent *t* test (2-tailed) or chi-square test as appropriate.

For eta-squared (η²) small effect sizes correspond to η²=0.01 (1% of variance explained), medium η²=0.06, and large η²=0.14. For Hedges *g* and Cohen *d*, small effect sizes correspond to 0.20, medium to 0.50, and large to 0.80 [54]. Within the moderator analysis, standardized beta coefficients serve as effect sizes.

### Analysis of Pre-Post Outcomes

We conducted between-group and within-group analyses to evaluate the effects of the interventions. For between-group comparisons, we used analysis of covariance with postintervention scores as dependent variables, intervention group (mentalization vs mindfulness) as the independent variable, and preintervention scores as covariates. This approach accounts for baseline differences and provides adjusted postintervention means for each group.

For within-group analyses, we conducted paired *t* tests (2-tailed) to evaluate pre-post changes within each intervention arm. Cohen *d* was calculated to quantify the magnitude of change, using the SD of the difference scores as the denominator, as recommended for paired designs.

Two trained researchers independently coded responses from the participant feedback survey using qualitative content analysis [55] to identify participants’ perceptions and suggestions for improvement. Coders developed a coding scheme and systematically applied codes to text segments. The researchers then conducted pattern analysis to identify emergent themes within the coded data and synthesized findings both within and across participant groups.

### EMA Data Analysis

To analyze the EMA data, we used a linear mixed-effects modeling approach that accounted for the hierarchical structure of repeated measurements nested within participants (31). For each EMA item, we fitted the following model: outcome_ij = β₀ + β₁(treatment)+β₂(group)+β₃(treatment×group)+ u₀ᵢ+ε_ij, where outcome_ij represents the EMA item for participant i at measurement occasion j, β₀ is the overall intercept, β₁ represents the main effect of treatment condition (ie, baseline and intervention phase), β₂ represents the main effect of group (mentalization and mindfulness), and β₃ represents the interaction between treatment and group. The model included a random intercept for each participant (u₀ᵢ) to account for between-person variability in baseline levels of the outcome. To address the temporal dependency in the repeated measurements, we specified a first-order autoregressive correlation structure with a 0.01 initial correlation value. The models were estimated using restricted maximum likelihood.

To quantify intervention effects, we calculated standardized mean difference effect sizes. We calculated both within-group effect sizes to evaluate baseline-intervention phase changes for each group separately and between-group effect sizes to compare treatment effects across groups. For within-group effect sizes, we calculated how much the treatment affected each group individually. For between-group comparisons, we calculated the differences in treatment effects when comparing one group to another.

All analyses were conducted in R [56] version 4.0.3 (R Foundation for Statistical Computing), using the “*nlme*” package [57] for model fitting and the “*scdhlm*” package [58] for effect size calculations. Full model results, including fixed and random effects estimates and model fit statistics, are available in the OSF project.

### Statistical Assumptions

Statistical assumptions were verified prior to analysis.

The homogeneity of regression slopes assumption was met for all analyses of covariance models. Normality of residuals was satisfied for primary outcomes. However, the LEE showed deviations from normality and was transformed (log transformation). Linearity assumptions were adequately met across all models.

Linear mixed-effects models met linearity, autocorrelation structure, and outlier assumptions. Level-1 residuals showed non-normality due to moderate skewness typical of affect measures, but multilevel models are robust to such violations. Random effects were appropriately specified and normally distributed.

### Ethical Considerations

Ethical approval was granted by the University College London Research Ethics Department (26261/00). All participants were provided with details of the study, confirmed eligibility, and provided informed written consent (see Checklist 1 for informed consent documentation). Participants were quasi-anonymous, personal identifiable information was collected at screening (and stored separately and securely), and a participant ID allocated thereafter (ie, the study data were deidentified). All participants (including those who chose to withdraw partway through) were entered into a prize draw to win a £50 (US $64) voucher. They received £20 (US $25) if they reached 60% completion of the EMA at the end of the study.

## Results

### Participant Flow

#### Recruitment

Participants were recruited between March and December 2024, with final follow-up assessments completed by February 2025. The target recruitment rate of 10 participants per month was met overall, although recruitment slowed during the summer months due to seasonal variation.

#### Baseline Data

Table 1 shows the descriptive statistics for baseline demographic characteristics and clinical measures for all participants randomized to each arm and the results of statistical comparisons between arms. There were no statistically significant differences between intervention arms at baseline on any of the variables measured. Participants ranged from 18 to 61 years, with a mean age of 23.50 (SD 6.56) years. The sample was skewed toward participants being female and predominantly self-identified as Asian or Asian British and White.

**Table 1.** Baseline characteristics of participants by trial arm.

Characteristic	Mentalization (n=37)	Mindfulness (n=38)	*P* value
Age (years)			.49
Mean (SD)	24.5 (8.23)	23.4 (5.09)	
Median (IQR)	22.0 (19.0-26.0)	22.0 (19.25-26.5)	
Sex, n (%)			.96
Male	7 (19)	6 (16)	
Female	30 (81)	32 (84)	
Ethnicity, n (%)			.88
Asian or Asian British	21 (57)	24 (63)	
Black or Black British	1 (3)	1 (3)	
Mixed or multiple	3 (8)	1 (3)	
White	9 (24)	9 (24)	
Other ethnic group	3 (8)	3 (8)	
GAD-7^a^			.21
Mean (SD)	12.3 (3.58)	11.1 (4.48)	
Median (IQR)	11.0 (10.00-15.0)	10.0 (8.25-13.0)	
PHQ-9^b^			.58
Mean (SD)	12.1 (3.80)	11.5 (4.40)	
Median (IQR)	11.0 (9.00-13.0)	11.0 (9.00-14.0)	
GAD^c^ clinical cutoff, n (%)			.12
Subclinical	1 (3)	6 (16)	
Caseness	36 (97)	32 (84)	
MDD^d^ clinical cutoff, n (%)			.67
Subclinical	10 (27)	13 (34)	
Caseness	27 (73)	25 (66)	
LEE^e^			.33
Mean (SD)	17.3 (12.8)	14.5 (12.4)	
Median (IQR)	14.0 (8.00-27.0)	10.5 (6.00-18.5)	
PID-5-BF^f^			.99
Mean (SD)	1.23 (0.47)	1.23 (0.43)	
Median (IQR)	1.26 (0.92-1.52)	1.22 (0.94-1.51)	
RFQ-8^g^			.61
Mean (SD)	3.66 (1.04)	3.53 (1.12)	
Median (IQR)	4.00 (2.75-4.63)	3.38 (2.66-4.25)	

a GAD-7: Generalized Anxiety Disorder-7.

b PHQ-9: Patient Health Questionnaire-9.

c GAD: generalized anxiety disorder.

d MDD: major depressive disorder.

e LEE: Level of Expressed Emotion.

f PID-5-BF: Personality Inventory for DSM-5 Brief Form.

g RFQ: Reflective Functioning Questionnaire.

Participants, on average, had moderate symptoms of depression and anxiety. All participants met caseness for either depression or generalized anxiety, while not significantly different (*P*=.12), 26 (70.3%) participants met caseness for both disorders in the mentalization arm and 19 (50%) participants in the mindfulness arm. On average, PID-5-BF scores suggest that participants had elevated personality traits at a level consistent with those diagnosed with personality disorder [59-61]

### Feasibility Outcomes

#### Recruitment

**Figure 1.**
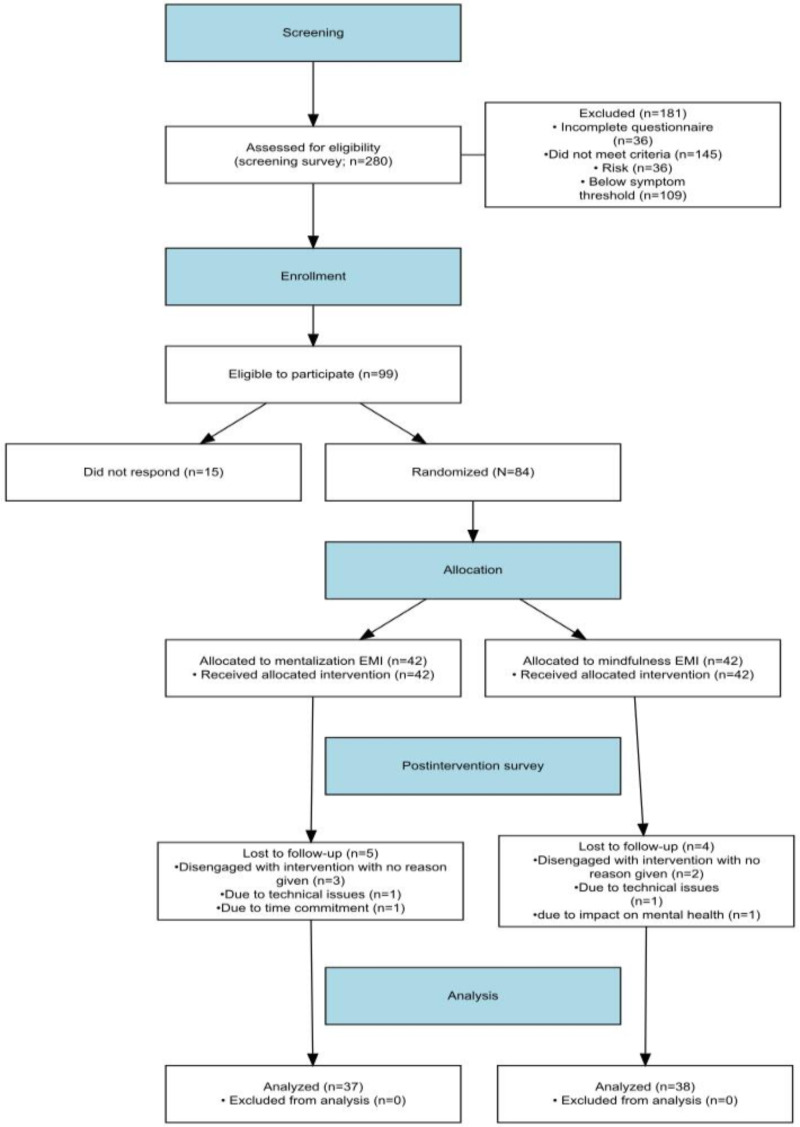
A CONSORT (Consolidated Standards of Reporting Trials) flow diagram showing participant flow through each stage of the pilot trial. EMI: ecological momentary interveintervention.

A total of 280 individuals were assessed for eligibility, of whom 99 (35%) individuals met inclusion criteria. A total of 84 (30%) individuals provided informed consent and were randomized (42 to each intervention). Figure 1 shows the complete participant flowchart through the trial.

#### Retention

Of those randomized, 5 (11.9%) participants disengaged from the mentalization EMI and 4 (9.5%) participants from the mindfulness EMI. Reported reasons for withdrawal included technical difficulties (n=3) and time constraints (n=1). For the remaining cases, reasons were not provided (n=3). One participant in the mindfulness condition withdrew due to perceived negative effects on their mental health, specifically an increase in body image-related distress linked to the intervention’s focus on bodily awareness. This was the only adverse reaction reported during the trial. Overall, 75 (89.2%) participants completed the intervention and final assessment.

A comparison between completers and noncompleters on baseline demographic and clinical variables revealed a statistically significant difference in age only (*t*_30.82_=−3.48; *P*<.001; *d*=0.40). Participants who withdrew (mean 20.11, SD 2.26) were, on average, 3.78 (SE 1.52) years younger than those who completed the study (mean 23.91, SD 6.80). No other significant differences were observed.

### Intervention Delivery and Adherence

Participants demonstrated strong adherence to the EMA protocol, completing an average of 98.21 (SD 12.69) EMAs throughout the study period, which represented 87.69% (SD 11.33%) of all scheduled assessments. Completion rates ranged from 57% to 100% across participants. When comparing the 2 intervention groups, adherence to the EMA protocol was comparable, with the mentalization group completing slightly more assessments, mean 99.32 (SD 11.61) than the mindfulness group, mean 97.13 (SD 13.73), though this difference was not statistically significant (*t*_73_ =0.746; *P*=.46; *d*=0.17).

Regarding EMIs, the intervention was triggered at similar rates across both groups, namely mentalization (79.4%) and mindfulness (78.6%). Both groups received comparable exposure to intervention opportunities. The mindfulness group showed slightly higher engagement (21.9%) compared to the mentalization group (18.3%). This represents the percentage of times participants actively engaged with the intervention when prompted. Participants completed an average of 15.60 (SD 22.74) EMI prompts throughout the study. There was wide variability in EMI completion rates, ranging from 0% to 100% in both groups. At the participant level, the average count was 14.4 (SD 23.0) interventions completed in the mentalization group and 16.8 (SD 22.7) in the mindfulness group. Maximum engagement for any participant was 95 (mentalization) and 90 (mindfulness) interventions.

### Acceptability Outcomes

**Figure 2.**
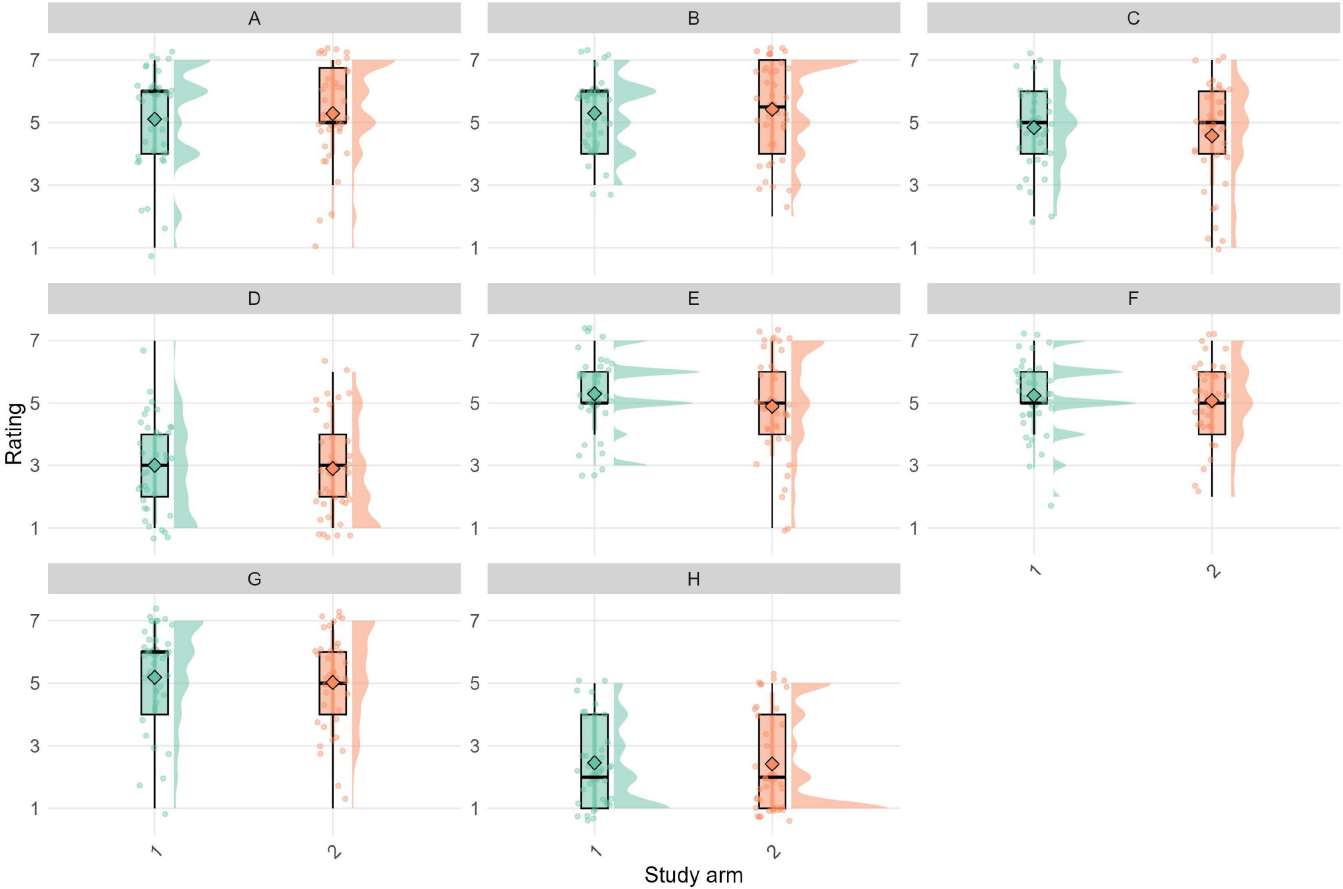
Acceptability question ratings for each group: (A) ease of engagement with the intervention; (B) ease of response to ecological momentary assessment (EMA); (C) impact on thinking about situations; (D) message intrusiveness; (E) awareness of mood and behavior; (F) overall enjoyment; (G) likelihood of recommending the intervention; and (H) time burden.

Figure 2 presents answers to the quantitative feedback questionnaire completed by the 75 participants who completed the study. Overall, participant ratings (on a 7-point Likert scale) related to app usability and experience were positive, with favorable scores for ease of engagement (mean 5.20, SD 1.57), ease of response (mean 5.36, SD 1.36), overall enjoyment (mean 5.15, SD 1.24), and likelihood of recommending the intervention (mean 5.11, SD 1.56).

There were no statistically significant differences between intervention groups on any questionnaire items (all *P*>.05). The mentalization group reported slightly higher scores on awareness of mood and behavior (mean 5.30, SD 1.20) compared to the mindfulness group (mean 4.89, SD 1.72; *t*_66.2_=1.18; *P*=.24; *d*=0.27) and a marginally higher impact on thinking about situations (mean 4.84, SD 1.32 vs mean 4.58, SD 1.69; *t*_69.9_=0.74; *P*=.46; *d*=0.17). The mindfulness group reported slightly higher ease of engagement with the intervention (mean 5.29, SD 1.54) than the mentalization group (mean 5.11, SD 1.61; *t*_72.6_=−0.50; *P*=.62; *d*=−0.12) and slightly higher ease of response to the EMA (mean 5.42, SD 1.52 vs mean 5.30, SD 1.20; *t*_70.0_=−0.39; *P*=.70; *d*=−0.09).

Participants across both groups reported low scores for message intrusiveness (mentalization: mean 3.00, SD 1.49; mindfulness: mean 2.89, SD 1.54; *t*_73.0_=0.30; *P*=.76; *d*=0.07) and time burden (mentalization: mean 2.46, SD 1.39; mindfulness: mean 2.42, SD 1.60; *t*_72.0_=0.11; *P*=.91; *d*=0.03), suggesting that participants did not find the level of engagement required overly intrusive or demanding.

Both groups reported similarly high levels of overall enjoyment (mentalization: mean 5.24, SD 1.19; mindfulness: mean 5.08, SD 1.30; *t*_72.7_=0.57; *P*=.57; *d*=0.13) and both groups were likely to recommend it to others (mentalization: mean 5.19, SD 1.60; mindfulness: mean 5.03, SD 1.53; *t*_72.7_=0.45; *P*=.65; *d*=0.10).

Participants’ qualitative feedback (n=32, 42.67% of study completers) was thematically coded (see OSF project). The mentalization group was significantly more likely to provide qualitative feedback than the mindfulness group (21 vs 11 participants; *χ*²_1_=5.9; *P*=.02).

Participants reported that the intervention enhanced awareness of internal states (n=12, 16%) and was user-friendly (n=7, 9%) and enjoyable (n=7, 9%). The mentalization group more frequently reported increased mental state awareness compared to the mindfulness group (n=14, 19% vs n=7, 9%).

Common criticisms included (1) interventions not being contextually relevant (n=12, 16%), (2) EMA prompts being too frequent (n=10, 13%), (3) confusing or irrelevant prompts (n=10, 13%), and (4) repetitive prompts (n=5, 6%). The mentalization group more often reported intervention relevance issues than the mindfulness group (15% vs 8%).

### Suggestions for Improvement

Individual suggestions included modifying EMA response options (n=2), adjusting prompt timing based on schedule changes (n=1), increasing compensation (n=1), and adding functionality to record supportive interactions (n=1).

### Potential Effectiveness Outcomes

#### Between-Group Differences

Comparative analyses revealed no statistically significant differences between the mentalization and mindfulness interventions on any pre-post outcome measure when controlling for baseline scores. Specifically, no significant differences were found for anxiety (*t*_72_=–0.49; *P*=.63; η²=0.003), depression (*t*_72_=–0.61; *P*=.54; η²=0.005), overall EE (*t*_72_=–0.002; *P*=.99; η²<0.001), or ineffective mentalizing (*t*_72_=0.62; *P*=.54; η²=0.005).

Effect sizes for all between-group comparisons were notably small (all η²<0.03), suggesting minimal practical differences between the interventions across all pre-post measured outcomes.

When comparing the differential effects between the mindfulness and mentalization groups on the EMA items, mindfulness demonstrated significant between-group effects over mentalization for assertiveness (*g*=0.265, 95% CI 0.096-0.434) and support (*g*=0.209, 95% CI 0.027-0.397).

#### Within-Group Changes

##### Primary Outcomes

###### Pre-Post Data

Both interventions showed significant improvements on several outcome measures. In the mentalization group, significant reductions were observed in anxiety symptoms (GAD-7: pre mean 12.30, SD 3.58; post mean 8.19, SD 4.25; *t*_36_=–4.94; *P*<.001; *d*=–0.81, 95% CI –1.15 to 0.47) and depression symptoms (PHQ-9: pre mean 12.05, SD 3.80; post mean 7.87, SD 4.29; *t*_36_=−4.24; *P*<.001; *d*=−0.70, 95% CI −1.03 to −0.37).

Similarly, the mindfulness group demonstrated significant reductions in anxiety (GAD-7: pre mean 11.11, SD 4.48; post mean 7.45, SD 4.30; *t*_37_=−4.16; *P*<.001; *d*=–0.68, 95% CI −1.01 to −0.35) and depression (PHQ-9: pre mean 11.53, SD 4.40; post mean 7.32, SD 3.86; *t*_37_=−4.41; *P*<.001; *d*=−0.72, 95% CI −1.05 to−0.39).

We also estimated changes in clinical caseness from pre- to postintervention for a diagnosis of depression or generalized anxiety disorder, an indicator of clinically significant change. In the mentalization group, prior to the intervention, 36 (97.3%) participants met criteria for generalized anxiety disorder (GAD) caseness, while 1 (2.7%) patient was subclinical. Postintervention, 16 (43.2%) participants showed improvement, no longer meeting caseness, while 21 (56.8%) participants exhibited no change. Notably, no participants worsened under this treatment approach (0, 0%). For depression, preintervention, 27 (73%) participants met criteria for depression caseness, while 10 (27%) participants were subclinical. 17 (45.9%) participants showed improvement, no change in 18 (48.6%) participants, and worsening in 2 (5.4%) participants.

For the mindfulness group (n=38), prior to intervention, 32 (84.2%) participants met criteria for GAD caseness while 6 (15.8%) participants were subclinical. A total of 17 (44.7%) participants improved, 19 (50.0%) participants showed no change, and 2 (5.3%) participants worsened. For depression, prior to the intervention, 25 (65.8%) participants met criteria for depression caseness while 13 (34.2%) participants were subclinical. Overall, 16 (42.1%) participants improved, 20 (52.6%) participants showed no change, and 2 (5.3%) participants worsened.

Both intervention approaches demonstrated clinically significant change in reducing symptoms below diagnostic thresholds, with the McNemar tests confirming significant changes in patient status across all conditions (all *P*≤.002).

###### EMA Data

**Figure 3.**
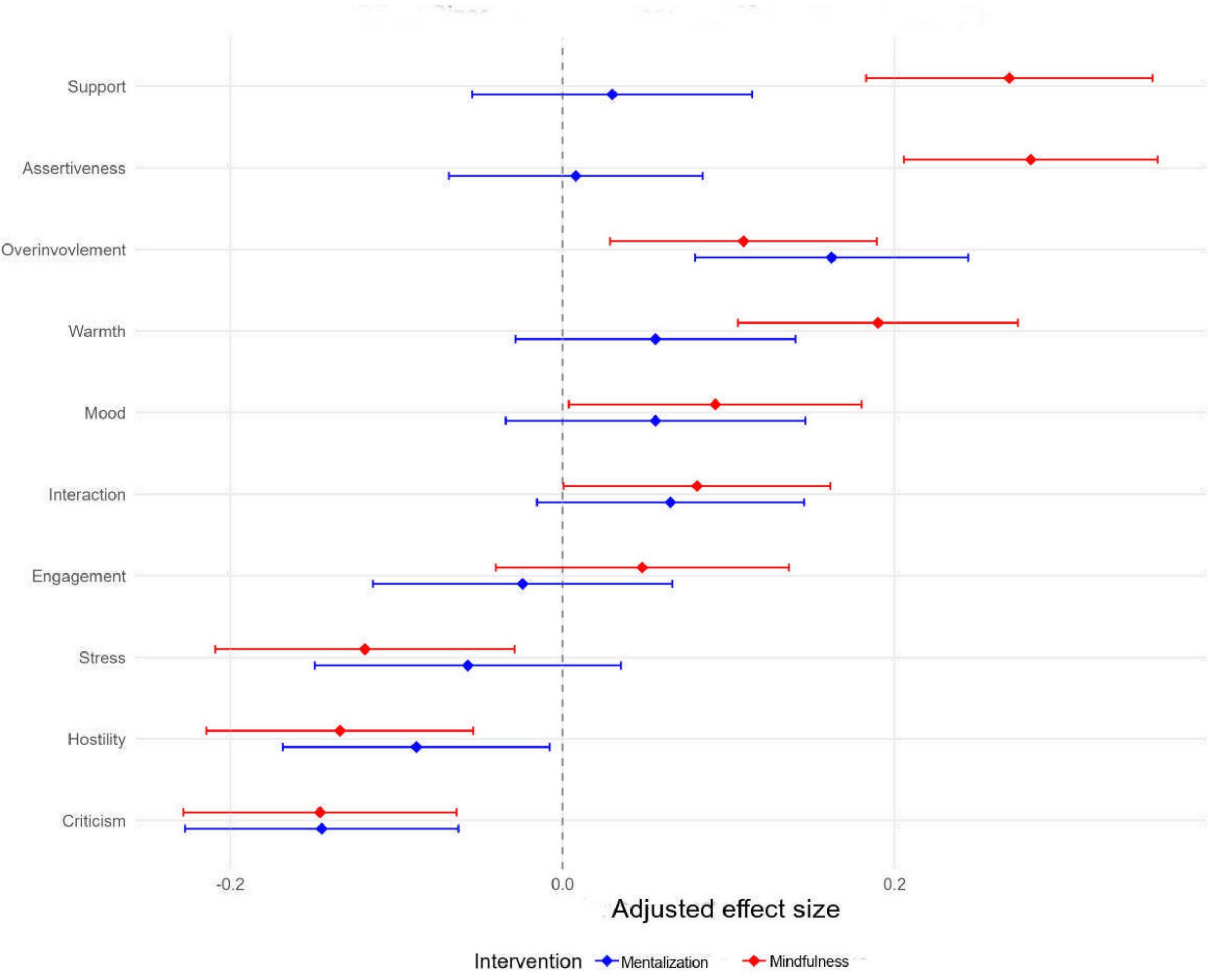
Forest plot of effect sizes by intervention group and outcome

The analysis of EMA data revealed several significant effects within each intervention group (see Figure 3). In the mentalization group, significant improvements were observed with a reduction in perceived criticism (*g*=−0.145, 95% CI −0.227 to −0.063), hostility (*g*=0.088, 95% CI −0.168 to −0.007), and an increase in overinvolvement (*g*=0.162, 95% CI 0.080-0.244).

The mindfulness group demonstrated more widespread significant changes. Participants showed reduced perceived criticism (*g*=−0.146, 95% CI −0.228 to −0.064), hostility (*g*=−0.134, 95% CI −0.214 to −0.054), and stress (*g*=−0.119, 95% CI −0.106 to −0.274). In addition, the mindfulness group exhibited significant improvements in assertiveness (*g*=0.282, 95% CI 0.206-0.358), support (*g*=0.269, 95% CI 0.183-0.355), warmth (*g*=0.190, 95% CI 0.105-0.274), overinvolvement (*g*=0.109, 95% CI 0.029-0.189), mood (*g*=0.092, 95% CI 0.004-0.180) and interaction (*g*=0.081, 95% CI 0.001-0.161).

Engagement showed no significant within-group changes in either intervention group.

### Secondary Outcomes

Exploratory analysis investigated the influence of the baseline RFQ-8, PID-5-BF, LEE, on outcomes.

Neither group showed significant changes in perceived EE (mentalization: LEE pre mean 17.32, SD 12.76; post mean 14.57, SD 10.38; *t*_36_=–1.81; *P*=.08; *d*=–0.30; mindfulness: pre mean 14.47, SD 12.39; post mean 13.00, SD 9.83; *t*_37_=–0.97; *P*=.33; *d*=–0.16).

Ineffective mentalizing increased slightly in both groups but was only statistically significant in the mindfulness group (mentalization: RFQ-8 pre mean 3.66, SD 1.04; post mean 3.96, SD 0.89; *t*_36_=1.68; *P*=.10; *d*=0.28; mindfulness: pre mean 3.53, SD 1.12; post mean 4.05, SD 1.04; *t*_37_=2.80; *P*=.008; *d*=0.455).

We investigated whether baseline measures predicted or moderated primary outcome intervention effects. When added to the PHQ-9 model, there was a significant interaction between intervention arm and baseline RFQ-8 scores (β=−2.05; *t*_70_=−2.40; *P*=.02), indicating that the effect of hypomentalizing on intervention outcomes differed significantly between groups. In the mentalization group, higher RFQ-8 scores (indicating poorer mentalizing) were associated with worse depression outcomes (β=1.91; *t*_70_=2.94; *P*=.004). In contrast, this relationship was slightly reversed in the mindfulness group (β=−0.15), suggesting that participants with poorer mentalizing abilities responded better to the mindfulness intervention relative to the mentalization intervention.

Comparison of nested models confirmed that the addition of the RFQ-8×treatment arm interaction significantly improved model fit (*F*_1, 70_=5.74; *P*=.02). The interaction pattern indicated a crossover effect at approximately 3.4, with mentalization predicting better outcomes below this threshold (ie, for those with less impaired mentalizing capacity) and mindfulness predicting better outcomes above this threshold (for those with more impaired mentalizing capacity).

There was a marginally significant main effect of RFQ-8 on posttreatment anxiety in the mentalization group (β=1.29; *t*_70_=1.94; *P*=.06), suggesting a potential trend whereby poorer mentalizing abilities predicted worse anxiety outcomes. However, the interaction between treatment arm and RFQ-8 was not significant (β=−0.11; *t*_70_=−0.12; *P*=.90).

Preintervention LEE scores (β=−0.10; *P*=.88 for PHQ-9; β=0.63; *P*=.40 for GAD-7) and their interaction with treatment arm (β=0.433; *P*=.42 for PHQ-9; β=−0.799, *P*=.46 for GAD-7) did not significantly predict postintervention scores. Similarly, preintervention PID-5-BF scores (β=0.17; *P*=.67 for PHQ-9; β=−0.08; *P*=.83 for GAD-7) and their interaction with treatment arm (β=0.06; *P*=.87 for PHQ-9; β=0.16; *P*=.67 for GAD-7) did not significantly predict postintervention scores.

Given the wide variation in EMI adherence rates, we also estimated the effect of intervention dose on clinical outcomes. The frequency of EMI adherence with either intervention did not meaningfully impact depression (main effect: β=−0.033; *P*=.27; interaction: (β=0.008; *P*=.86) or anxiety (main effect: β=−0.018; *P*=.56; interaction: β=0.010; *P*=.81) outcomes.

## Discussion

### Principal Findings

This pilot randomized controlled trial aimed to assess the feasibility, acceptability, and preliminary effects of mindfulness and mentalization-based microinterventions delivered via smartphone, targeting perceived EE, for individuals experiencing common mental health problems. To our knowledge, this is the first EMI addressing mentalizing specifically. Our results indicate that both interventions were feasible, with strong recruitment and retention rates and high adherence to the EMI protocol. Participants reported high levels of acceptability, finding the interventions easy to use, helpful, and not burdensome. Preliminary outcome data revealed that both the mentalization and mindfulness interventions were associated with significant within-group reductions in symptoms of anxiety and depression from pre- to postintervention, with a significant proportion of participants no longer meeting caseness criteria for generalized anxiety or major depression postintervention. There were no statistically significant differences between the mentalization and mindfulness groups on postintervention symptom scores.

This pilot trial provides valuable insights into the practical implementation of automated mindfulness and mentalization-based EMIs for individuals with common mental health problems. The high retention and adherence rates are particularly encouraging, suggesting that despite the intensive nature of EMA, which can lead to high dropout [62], participants found the overall study protocol manageable and were motivated to see it through. This suggests that individuals with clinically significant anxiety and depression are able and willing to engage with this delivery format over a sustained period.

The quantitative feedback indicated high levels of acceptability of the intervention delivered via the app. Participants gave favorable ratings for ease of engagement, ease of response, overall enjoyment, and likelihood of recommending the app. Crucially for an EMI delivered in daily life, participants reported low levels of perceived intrusiveness and time burden, supporting the notion that these microinterventions can be integrated into daily routines without being overly disruptive. Participants highlighted that the interventions enhanced their awareness of internal states and were user-friendly and enjoyable. The mentalization group specifically reported increased mental state awareness more frequently.

The high retention and adherence rates appear promising and align with the general potential of EMIs to increase accessibility and reduce treatment gaps. While direct comparisons are challenging due to variations in populations, intervention content, duration, and delivery methods, studies of other digital mental health interventions and EMIs report variable adherence rates [63,64], making the strong engagement observed here a notable positive finding. The high acceptability ratings, particularly regarding low intrusiveness, support the theoretical advantage of EMIs delivered via smartphones in integrating support into daily life. Finally, the report of one unintended harm in the mindfulness arm, though infrequent, highlights the critical importance of safety protocols and careful content design even in seemingly low-risk digital interventions, particularly when focusing on potentially sensitive areas like the body, and is a relatively common adverse effect reported in studies of mindfulness interventions [65].

This pilot offers insights into the potential clinical impact of these EMIs. Both the mentalization and mindfulness intervention groups demonstrated moderate to large significant within-group reductions in symptoms of both anxiety and depression. The within-group improvements observed here align with research indicating that EMIs can lead to symptom reduction in depression and anxiety [7]. While direct comparisons to effect sizes in traditional or other digital interventions are challenging due to differences in methodology, population, and intervention intensity, the observed effect sizes for symptom reduction are within a range consistent with mindfulness approaches in other formats [66] and higher than those in other mental health EMIs [67], suggesting that the observed signal is substantial enough to warrant further investigation in a controlled trial. Analyses of clinical caseness showed that a significant proportion of participants in both groups no longer met diagnostic thresholds postintervention, representing a clinically significant change in patient status from preintervention.

Despite the promising within-group improvements, comparative analyses revealed no statistically significant differences between the mentalization and mindfulness groups on primary outcome measures like anxiety or depression symptom reduction. The findings suggest that, on average, both intervention types may offer similar benefits. However, the potential moderating role of baseline mentalizing capacity (hypomentalizing) on depression outcomes suggested that those with poorer mentalizing abilities responded relatively better to the mindfulness intervention. Lower baseline mentalizing ability may have hindered engagement with mentalizing exercises due to their cognitive and emotional demands. This result may indicate that those characterized by more marked hypomentalizing or less effective mentalizing benefit more from a soothing and emotion-regulatory approach (which might then allow for more mentalizing to become possible only after that has been achieved), while those with less pronounced mentalizing difficulties can engage with a mentalizing process in a way that can help alleviate symptoms. However, there are also concerns about the RFQ-8 primarily measuring hypomentalization of the self [68], while individuals with interpersonal difficulties are more likely to struggle with hypermentalization of others [69,70]. Pre-existing levels of these specific psychological capacities might influence which type of intervention is more beneficial, a finding that warrants further investigation.

The analysis of momentary EMA data provided more granular insight about potential mechanisms of change occurring during the intervention phase. While both groups showed a significant reduction in perceived criticism and hostility and an increase in overinvolvement, the mindfulness group demonstrated a broader range of significant improvements in momentary experiences. These momentary changes hint at how the interventions might operate in daily life. Mindfulness, by fostering present-moment awareness and acceptance, may facilitate less reactive responses to stressful interpersonal triggers and increase positive interpersonal engagement [71]. Mentalizing, by encouraging reflection on mental states, might directly alter interpretations of others’ behavior (eg, reducing perceived criticism) and potentially lead to different interaction patterns [30]. The qualitative feedback also supports a potential mechanism of increased self-awareness, which participants across groups highlighted. The increase in overinvolvement, in line with most positive variables, is an interesting finding, as theoretically it is a component of EE. However, there are cross-cultural differences [16], and it hints that this variable may be interpreted differently by this cohort.

This study presents several notable strengths. Methodologically, the use of a randomized controlled trial design, even for a pilot study with a 1:1 allocation ratio, helps to reduce selection bias and allows for a more rigorous comparison between the 2 intervention arms. The integration of EMA is a key strength, enabling the collection of real-time data on momentary experiences, behaviors, and symptoms within participants’ natural environments, which is particularly valuable for understanding the dynamic nature of mental health and potential mechanisms of change. The trial was also preregistered and data and code are openly available, increasing transparency and reproducibility.

These findings are subject to several important limitations. The small sample size (n=84 randomized) inherently limits the statistical power to detect significant differences, particularly when evaluating differences across a number of measures. Preliminary efficacy findings, while showing promising within-group trends, should be interpreted with caution. While there were comparison arms, and each individual acted as their own control (baseline and intervention phases), there was no passive control arm for assessment of EMA outcomes. An arm with EMA only would have been a useful addition, as EMA alone is likely to lead to greater self-awareness (mentalizing of self) and bring about changes in mood [64,72]. In addition, while the probability of spontaneous remission during such a short window is low [73,74], it cannot be ruled out. A fully powered trial, designed with mediation analysis in mind, is required to formally test the hypothesis that changes in perceived EE mediate the effects of these interventions on clinical outcomes.

This EMI approach uses a “less is more” philosophy, focusing on developing specific skills through repeated practice embedded in daily life. However, implementation revealed significant variability in completion rates (0%‐100%) with no clear dose-response relationship, complicating effectiveness assessment. A notable challenge emerged in the disconnect between EMA assessment completion and actual engagement with the triggered microinterventions. Participants often responded to assessment prompts while bypassing the therapeutic content. Qualitative feedback suggested several potential explanations: (1) interventions sometimes lacked contextual relevance (particularly in the mentalization group), (2) prompts became overly frequent or repetitive, or (3) participants struggled to engage with specific content at the moment of delivery. The simplicity of the strategies may also influence engagement patterns. Once learned, participants might implement strategies independently upon receiving prompts, without needing to follow the step-by-step guidance in the app each time. This self-directed app could explain intervention effectiveness despite lower formal completion rates. While EMA alone might produce some change effects, the observed differences between intervention groups on EMA items suggest specific intervention impacts beyond assessment effects. Postintervention qualitative interviews would have provided valuable insights into these engagement patterns and helped distinguish between assessment and intervention effects. Future refinements should address these engagement challenges by modifying response options, adjusting prompt timing, and ensuring contextual relevance to maximize therapeutic engagement.

The short intervention duration (21 days of EMI) means the findings capture only immediate postintervention changes and do not provide insight into the long-term sustainability of effects. Lack of long-term follow-up is a common limitation of pilot studies and restricts understanding of enduring clinical impact. Although retention was high overall, dropout did occur, and those who dropped out were significantly younger, suggesting potential differences in engagement based on age. The sample population, primarily recruited from a university setting and skewed toward younger adults, females, and those self-identified as Asian or Asian British, and White ethnicity, limits the generalizability of the findings to broader populations with common mental health problems [75]. Elevated maladaptive personality traits within the sample highlight the need to take an integrative approach to personality and psychopathology [76] and consider interpersonal and personality functioning in common mental health disorders [77].

The presence of incentives likely contributed to the high retention and adherence rates observed. The removal of elements like financial incentives, intensive monitoring and reminders by researchers, and direct human risk management in a routine application setting could significantly impact user retention. The motivation levels of users to participate may drop, resulting in inconsistent engagement patterns and insufficient engagement duration. The precise activation of interventions based on data combined with the rigorous assessment schedule proves beneficial for research; however, it demands modification to enhance user experience and maintain engagement outside research settings.

Further work is required to optimize the threshold for triggering the interventions. The current design fits within the Multiphase Optimization Strategy framework [78], aiming to refine the selected microinterventions and investigate their optimal levels. In developing these interventions, more emphasis needs to be placed on the dynamic tailoring of interventions based on an individual’s evolving context and needs [79,80]. A significant hurdle lies in the selection and integration of existing empirical, theoretical, and practical evidence that can effectively guide the design and development of these just-in-time adaptive interventions [81,82]. Methods, such as statistical process control and exponentially weighted moving average procedures, may be more appropriate for identifying change [83] but also reinforcement learning models [84] Accurately assessing an individual’s receptivity to an intervention at any given moment presents another challenge, as factors such as their current location, emotional state, and social environment can influence their willingness and ability to engage.

### Conclusion

These positive indicators of feasibility and acceptability, alongside promising preliminary effectiveness data, suggest that both mindfulness and mentalization-based EMIs are viable approaches for individuals with common mental health problems and warrant further evaluation, ideally in the format of a sequential multiple assignment randomized trial, which would allow for the development and evaluation of the effectiveness of multiple adaptive treatment strategies in addition to evaluations of single interventions at multiple stages [85]. Future iterations of these interventions should consider refinements, including optimizing the contextual relevance of the interventions, adjusting intervention prompt frequency through refined thresholds, and enhancing the clarity and variability of the prompts to avoid repetitiveness. This pilot study contributes to the literature by showing that short, theory-based microinterventions can be delivered in everyday settings as a feasible approach, which opens opportunities for further exploration into their potential to enhance psychological well-being across broader populations.

## Supplementary material

10.2196/79296 Checklist 1 CONSORT-EHEALTH (V1.6.1) checklist.

## Abbreviations

CONSORT-EHEALTH: Consolidated Standards of Reporting Trials of Electronic and Mobile Health Applications and Online Telehealth
EE: expressed emotion
EMA: ecological momentary assessment
EMI: ecological momentary intervention
GAD: generalized anxiety disorder
GAD-7: Generalized Anxiety Disorder Questionnaire-7
LEE: Level of Expressed Emotion
OSF: Open Science Framework
PHQ-9: Patient Health Questionnaire-9
PID-5-BF: Personality Inventory for DSM-5 – Brief Form
RFQ-8: Reflective Functioning Questionnaire-8
STORM: Skills Training On Risk Management
