# Supplementary material for: Smartphone-Based Mindfulness and Mentalization Ecological Momentary Interventions for Common Mental Health Problems: Pilot Randomized Controlled Trial
Source: JMIR Ment Health. 2025 Sep 30;12:e79296. doi: 10.2196/79296 (PMC12483342; doi:10.2196/79296)
Supplement: Checklist 1 [file mental-v12-e79296-s001.pdf]

CONSORT-EHEALTH (V 1.6.1) - Submission/Publication Form

The CONSORT-EHEALTH checklist is intended for authors of randomized trials evaluating web-based and Internet-based applications/interventions, including mobile interventions, electronic games (incl multiplayer games), social media, certain telehealth applications, and other interactive and/or networked electronic applications. Some of the items (e.g. all subitems under item 5 - description of the intervention) may also be applicable for other study designs.

The goal of the CONSORT EHEALTH checklist and guideline is to be
a) a guide for reporting for authors of RCTs,
b) to form a basis for appraisal of an ehealth trial (in terms of validity)

CONSORT-EHEALTH items/subitems are MANDATORY reporting items for studies published in the Journal of Medical Internet Research and other journals / scientific societies endorsing the checklist.

Items numbered 1., 2., 3., 4a., 4b etc are original CONSORT or CONSORT-NPT (non-pharmacologic treatment) items.
Items with Roman numerals (i., ii, iii, iv etc.) are CONSORT-EHEALTH extensions/clarifications.

As the CONSORT-EHEALTH checklist is still considered in a formative stage, we would ask that you also RATE ON A SCALE OF 1-5 how important/useful you feel each item is FOR THE PURPOSE OF THE CHECKLIST and reporting guideline (optional).

Mandatory reporting items are marked with a red *.
In the textboxes, either copy & paste the relevant sections from your manuscript into this form - please include any quotes from your manuscript in QUOTATION MARKS,
or answer directly by providing additional information not in the manuscript, or elaborating on why the item was not relevant for this study.

YOUR ANSWERS WILL BE PUBLISHED AS A SUPPLEMENTARY FILE TO YOUR PUBLICATION IN JMIR AND ARE CONSIDERED PART OF YOUR PUBLICATION (IF ACCEPTED).
Please fill in these questions diligently. Information will not be copyedited, so please use proper spelling and grammar, use correct capitalization, and avoid abbreviations.

DO NOT FORGET TO SAVE AS PDF _AND_ CLICK THE SUBMIT BUTTON SO YOUR ANSWERS ARE IN OUR DATABASE !!!

Citation Suggestion (if you append the pdf as Appendix we suggest to cite this paper in the caption):
Eysenbach G, CONSORT-EHEALTH Group
[illegible]
J Med Internet Res 2011;13(4):e126

You're editing your response. Sharing this URL allows others to also edit your response.

FILL IN A NEW RESPONSE

URL: http://www.jmir.org/2011/4/e126/
doi: 10.2196/jmir.1923
PMID: 22209829

ciaran.odriscoll@gmail.com Switch accounts

Not shared

Resubmit to save

* Indicates required question

Your name *

First Last

Ciaran O'Driscoll

Primary Affiliation (short), City, Country *

University of Toronto, Toronto, Canada

UCL, UK

Your e-mail address *

abc@gmail.com

c.odriscoll@gmail.com

Title of your manuscript *

Provide the (draft) title of your manuscript.

Feasibility and Acceptability of Mindfulness and Mentalisation smartphone based Ecological Momentary Interventions for Common Mental Health Problems: A Pilot Randomized Controlled Trial

You're editing your response. Sharing this URL allows others to also edit your response.

FILL IN A NEW RESPONSE

Name of your App/Software/Intervention *

If there is a short and a long/alternate name, write the short name first and add the long name in brackets.

MMEMI

Evaluated Version (if any)

e.g. "V1", "Release 2017-03-01", "Version 2.0.27913"

v1.0000

Language(s) *

What language is the intervention/app in? If multiple languages are available, separate by comma (e.g. "English, French")

English

URL of your Intervention Website or App

e.g. a direct link to the mobile app on app in appstore (itunes, Google Play), or URL of the website. If the intervention is a DVD or hardware, you can also link to an Amazon page.

N/A

URL of an image/screenshot (optional)

Your answer

You're editing your response. Sharing this URL allows others to also edit your response.

FILL IN A NEW RESPONSE

Accessibility *

Can an enduser access the intervention presently?

- ○ access is free and open
- ◉ access only for special usergroups, not open
- ○ access is open to everyone, but requires payment/subscription/in-app purchases
- ○ app/intervention no longer accessible
- ○ Other:

Primary Medical Indication/Disease/Condition *

e.g. "Stress", "Diabetes", or define the target group in brackets after the condition, e.g. "Autism (Parents of children with)", "Alzheimers (Informal Caregivers of)"

Common Mental Health Problems (Depression

Primary Outcomes measured in trial *

comma-separated list of primary outcomes reported in the trial

PHQ-9; GAD-7

Secondary/other outcomes

Are there any other outcomes the intervention is expected to affect?

EMA items, LEE, RFQ measures

You're editing your response. Sharing this URL allows others to also edit your response.

FILL IN A NEW RESPONSE

Recommended "Dose" *

What do the instructions for users say on how often the app should be used?

- (selected) Approximately Daily
- Approximately Weekly
- Approximately Monthly
- Approximately Yearly
- "as needed"
- Other:

Approx. Percentage of Users (starters) still using the app as recommended after 3 months *

- (selected) unknown / not evaluated
- 0-10%
- 11-20%
- 21-30%
- 31-40%
- 41-50%
- 51-60%
- 61-70%
- 71%-80%
- 81-90%
- 91-100%
- Other:

You're editing your response. Sharing this URL allows others to also edit your response.

FILL IN A NEW RESPONSE

Overall, was the app/intervention effective? *

- ◯ yes: all primary outcomes were significantly better in intervention group vs control
- ◯ partly: SOME primary outcomes were significantly better in intervention group vs control
- ◯ no statistically significant difference between control and intervention
- ◯ potentially harmful: control was significantly better than intervention in one or more outcomes
- ◯ inconclusive: more research is needed
- ◉ Other: control was within group (A/B design) intervention were sig on primar

Article Preparation Status/Stage *

At which stage in your article preparation are you currently (at the time you fill in this form)

- ◯ not submitted yet - in early draft status
- ◉ not submitted yet - in late draft status, just before submission
- ◯ submitted to a journal but not reviewed yet
- ◯ submitted to a journal and after receiving initial reviewer comments
- ◯ submitted to a journal and accepted, but not published yet
- ◯ published
- ◯ Other:

You're editing your response. Sharing this URL allows others to also edit your response.

FILL IN A NEW RESPONSE

Journal *

If you already know where you will submit this paper (or if it is already submitted), please provide the journal name (if it is not JMIR, provide the journal name under "other")

- ○ not submitted yet / unclear where I will submit this
- ○ Journal of Medical Internet Research (JMIR)
- ○ JMIR mHealth and UHealth
- ○ JMIR Serious Games
- ◉ JMIR Mental Health
- ○ JMIR Public Health
- ○ JMIR Formative Research
- ○ Other JMIR sister journal
- ○ Other:

Is this a full powered effectiveness trial or a pilot/feasibility trial? *

- ◉ Pilot/feasibility
- ○ Fully powered

Manuscript tracking number *

If this is a JMIR submission, please provide the manuscript tracking number under "other" (The ms tracking number can be found in the submission acknowledgement email, or when you login as author in JMIR. If the paper is already published in JMIR, then the ms tracking number is the four-digit number at the end of the DOI, to be found at the bottom of each published article in JMIR)

- ◉ no ms number (yet) / not (yet) submitted to / published in JMIR
- ○ Other:

You're editing your response. Sharing this URL allows others to also edit your response.

FILL IN A NEW RESPONSE

TITLE AND ABSTRACT

1a) TITLE: Identification as a randomized trial in the title

1a) Does your paper address CONSORT item 1a? *

I.e does the title contain the phrase "Randomized Controlled Trial"? (if not, explain the reason under "other")

- (selected) yes
- Other:

1a-i) Identify the mode of delivery in the title

Identify the mode of delivery. Preferably use “web-based” and/or “mobile” and/or “electronic game” in the title. Avoid ambiguous terms like “online”, “virtual”, “interactive”. Use “Internet-based” only if Intervention includes non-web-based Internet components (e.g. email), use “computer-based” or “electronic” only if offline products are used. Use “virtual” only in the context of “virtual reality” (3-D worlds). Use “online” only in the context of “online support groups”. Complement or substitute product names with broader terms for the class of products (such as “mobile” or “smart phone” instead of “iphone”), especially if the application runs on different platforms.

	1	2	3	4	5	
subitem not at all important	○	○	○	●	○	essential

Clear selection

Does your paper address subitem 1a-i? *

Copy and paste relevant sections from manuscript title (include quotes in quotation marks "like this" to indicate direct quotes from your manuscript), or elaborate on this item by providing additional information not in the ms, or briefly explain why the item is not applicable/relevant for your study

You're editing your response. Sharing this URL allows others to also edit your response.

FILL IN A NEW RESPONSE

1a-ii) Non-web-based components or important co-interventions in title

Mention non-web-based components or important co-interventions in title, if any (e.g., "with telephone support").

	1	2	3	4	5	
subitem not at all important	○	○	○	●	○	essential

Clear selection

Does your paper address subitem 1a-ii?

Copy and paste relevant sections from manuscript title (include quotes in quotation marks "like this" to indicate direct quotes from your manuscript), or elaborate on this item by providing additional information not in the ms, or briefly explain why the item is not applicable/relevant for your study

the co-interventions "Mindfulness and Mentalisation" are mentioned

1a-iii) Primary condition or target group in the title

Mention primary condition or target group in the title, if any (e.g., "for children with Type I Diabetes") Example: A Web-based and Mobile Intervention with Telephone Support for Children with Type I Diabetes: Randomized Controlled Trial

	1	2	3	4	5	
subitem not at all important	○	○	○	○	●	essential

Clear selection

Does your paper address subitem 1a-iii? *

Copy and paste relevant sections from manuscript title (include quotes in quotation marks "like this" to indicate direct quotes from your manuscript), or elaborate on this item by providing additional information not in the ms, or briefly explain why the item is not applicable/relevant for your study

You're editing your response. Sharing this URL allows others to also edit your response.

FILL IN A NEW RESPONSE

1b) ABSTRACT: Structured summary of trial design, methods, results, and conclusions

NPT extension: Description of experimental treatment, comparator, care providers, centers, and blinding status.

1b-i) Key features/functionalities/components of the intervention and comparator in the METHODS section of the ABSTRACT

Mention key features/functionalities/components of the intervention and comparator in the abstract. If possible, also mention theories and principles used for designing the site. Keep in mind the needs of systematic reviewers and indexers by including important synonyms. (Note: Only report in the abstract what the main paper is reporting. If this information is missing from the main body of text, consider adding it)

	1	2	3	4	5	
subitem not at all important	○	○	◉	○	○	essential

Clear selection

Does your paper address subitem 1b-i? *

Copy and paste relevant sections from the manuscript abstract (include quotes in quotation marks "like this" to indicate direct quotes from your manuscript), or elaborate on this item by providing additional information not in the ms, or briefly explain why the item is not applicable/relevant for your study

Participants experiencing common mental health problems were recruited online, from a University setting. Eligible participants were randomly allocated to fully automated mindfulness-based or mentalisation-based ecological momentary interventions via computer-generated randomisation. Participants were blind to the alternative intervention options. Outcomes were self-assessed through questionnaires. Primary outcomes were feasibility (recruitment, retention, adherence) and acceptability (satisfaction ratings, qualitative feedback). Secondary outcomes included changes in depression (PHQ-9) and anxiety (GAD-7) scores

You're editing your response. Sharing this URL allows others to also edit your response.

FILL IN A NEW RESPONSE

1b-ii) Level of human involvement in the METHODS section of the ABSTRACT

Clarify the level of human involvement in the abstract, e.g., use phrases like “fully automated” vs. “therapist/nurse/care provider/physician-assisted” (mention number and expertise of providers involved, if any). (Note: Only report in the abstract what the main paper is reporting. If this information is missing from the main body of text, consider adding it)

	1	2	3	4	5	
subitem not at all important	○	○	○	◉	○	essential

Clear selection

Does your paper address subitem 1b-ii?

Copy and paste relevant sections from the manuscript abstract (include quotes in quotation marks "like this" to indicate direct quotes from your manuscript), or elaborate on this item by providing additional information not in the ms, or briefly explain why the item is not applicable/relevant for your study

"fully automated mindfulness-based or mentalisation-based ecological momentary interventions"

You're editing your response. Sharing this URL allows others to also edit your response.

FILL IN A NEW RESPONSE

1b-iii) Open vs. closed, web-based (self-assessment) vs. face-to-face assessments in the METHODS section of the ABSTRACT

Mention how participants were recruited (online vs. offline), e.g., from an open access website or from a clinic or a closed online user group (closed usergroup trial), and clarify if this was a purely web-based trial, or there were face-to-face components (as part of the intervention or for assessment). Clearly say if outcomes were self-assessed through questionnaires (as common in web-based trials). Note: In traditional offline trials, an open trial (open-label trial) is a type of clinical trial in which both the researchers and participants know which treatment is being administered. To avoid confusion, use “blinded” or “unblinded” to indicated the level of blinding instead of “open”, as “open” in web-based trials usually refers to “open access” (i.e. participants can self-enrol). (Note: Only report in the abstract what the main paper is reporting. If this information is missing from the main body of text, consider adding it)

	1	2	3	4	5	
subitem not at all important	○	○	○	◉	○	essential

Clear selection

Does your paper address subitem 1b-iii?

Copy and paste relevant sections from the manuscript abstract (include quotes in quotation marks "like this" to indicate direct quotes from your manuscript), or elaborate on this item by providing additional information not in the ms, or briefly explain why the item is not applicable/relevant for your study

Yes. "Outcomes were self-assessed through questionnaires. " "Participants were blind to the alternative intervention options."

You're editing your response. Sharing this URL allows others to also edit your response.

FILL IN A NEW RESPONSE

1b-iv) RESULTS section in abstract must contain use data

Report number of participants enrolled/assessed in each group, the use/uptake of the intervention (e.g., attrition/adherence metrics, use over time, number of logins etc.), in addition to primary/secondary outcomes. (Note: Only report in the abstract what the main paper is reporting. If this information is missing from the main body of text, consider adding it)

	1	2	3	4	5	
subitem not at all important	○	○	○	◉	○	essential

Clear selection

Does your paper address subitem 1b-iv?

Copy and paste relevant sections from the manuscript abstract (include quotes in quotation marks "like this" to indicate direct quotes from your manuscript), or elaborate on this item by providing additional information not in the ms, or briefly explain why the item is not applicable/relevant for your study

Yes. Eighty-four participants were randomised (42 to each group). The intervention demonstrated good feasibility. The interventions demonstrated good feasibility with 89.2% retention rate and mean adherence of 87.69% across both groups. Acceptability ratings were positive, with favourable scores for ease of engagement (M = 5.20), overall enjoyment (M = 5.15), and likelihood of recommending the app (M = 5.11) on a 7-point scale. For primary outcomes, both groups showed significant within-group reductions in PHQ-9 and GAD-7 scores, with moderate to large effect sizes (d= -0.68 to -0.81), with no significant difference between groups. Both treatments demonstrated clinically significant effectiveness in reducing symptoms below diagnostic thresholds (42 - 46%). Mindfulness performed better on improving assertiveness and perceived support compared to mentalisation in the momentary EMA data. One unintended harm was reported in the mindfulness arm, none in the mentalisation arm.

You're editing your response. Sharing this URL allows others to also edit your response.

FILL IN A NEW RESPONSE

1b-v) CONCLUSIONS/DISCUSSION in abstract for negative trials

Conclusions/Discussions in abstract for negative trials: Discuss the primary outcome - if the trial is negative (primary outcome not changed), and the intervention was not used, discuss whether negative results are attributable to lack of uptake and discuss reasons. (Note: Only report in the abstract what the main paper is reporting. If this information is missing from the main body of text, consider adding it)

	1	2	3	4	5	
subitem not at all important	○	○	◉	○	○	essential

Clear selection

Does your paper address subitem 1b-v?

Copy and paste relevant sections from the manuscript abstract (include quotes in quotation marks "like this" to indicate direct quotes from your manuscript), or elaborate on this item by providing additional information not in the ms, or briefly explain why the item is not applicable/relevant for your study

This pilot trial suggests that both mindfulness and mentalisation-based ecological momentary interventions are feasible and acceptable for individuals with common mental health problems and warrant further evaluation.

INTRODUCTION

2a) In INTRODUCTION: Scientific background and explanation of rationale

You're editing your response. Sharing this URL allows others to also edit your response.

FILL IN A NEW RESPONSE

2a-i) Problem and the type of system/solution

Describe the problem and the type of system/solution that is object of the study: intended as stand-alone intervention vs. incorporated in broader health care program? Intended for a particular patient population? Goals of the intervention, e.g., being more cost-effective to other interventions, replace or complement other solutions? (Note: Details about the intervention are provided in "Methods" under 5)

	1	2	3	4	5	
subitem not at all important	○	○	○	◉	○	essential

Clear selection

Does your paper address subitem 2a-i? *

Copy and paste relevant sections from the manuscript (include quotes in quotation marks "like this" to indicate direct quotes from your manuscript), or elaborate on this item by providing additional information not in the ms, or briefly explain why the item is not applicable/relevant for your study

" The integration of mindfulness and mentalisation micro-interventions into smartphone-based EMIs presents a promising avenue for providing accessible and timely support to individuals with common mental health problems in their daily lives, precisely when they might be navigating situations involving perceived expressed emotion. This could complement other psychological approaches. "

2a-ii) Scientific background, rationale: What is known about the (type of) system

Scientific background, rationale: What is known about the (type of) system that is the object of the study (be sure to discuss the use of similar systems for other conditions/diagnoses, if appropiate), motivation for the study, i.e. what are the reasons for and what is the context for this specific study, from which stakeholder viewpoint is the study performed, potential impact of findings [2]. Briefly justify the choice of the comparator.

	1	2	3	4	5	
subitem not at all important	○	○	○	◉	○	essential

Clear selection

You're editing your response. Sharing this URL allows others to also edit your response.

FILL IN A NEW RESPONSE

Does your paper address subitem 2a-ii? *

Copy and paste relevant sections from the manuscript (include quotes in quotation marks "like this" to indicate direct quotes from your manuscript), or elaborate on this item by providing additional information not in the ms, or briefly explain why the item is not applicable/relevant for your study

this is outlined in paragraph 3, which explains the role of expressed emotion, and rationale for the mindfulness and mentalisation EMIs in paragraph 4 of the introduction.

2b) In INTRODUCTION: Specific objectives or hypotheses

Does your paper address CONSORT subitem 2b? *

Copy and paste relevant sections from the manuscript (include quotes in quotation marks "like this" to indicate direct quotes from your manuscript), or elaborate on this item by providing additional information not in the ms, or briefly explain why the item is not applicable/relevant for your study

Objectives:

· To determine the feasibility (e.g., recruitment rates, adherence, retention) and acceptability (e.g., usability, satisfaction) of delivering mindfulness and mentalization-based micro-interventions via smartphone EMIs to individuals experiencing common mental health problems.

· To investigate preliminary indications of efficacy by examining potential changes on relevant clinical outcomes.

METHODS

3a) Description of trial design (such as parallel, factorial) including allocation ratio

You're editing your response. Sharing this URL allows others to also edit your response.

FILL IN A NEW RESPONSE

Does your paper address CONSORT subitem 3a? *

Copy and paste relevant sections from the manuscript (include quotes in quotation marks "like this" to indicate direct quotes from your manuscript), or elaborate on this item by providing additional information not in the ms, or briefly explain why the item is not applicable/relevant for your study

This pilot randomized controlled trial used a parallel-group design with a 1:1 allocation ratio. The trial was exploratory in nature, with the primary aim of assessing the feasibility and acceptability of the EMIs prior to a definitive trial.

3b) Important changes to methods after trial commencement (such as eligibility criteria), with reasons

Does your paper address CONSORT subitem 3b? *

Copy and paste relevant sections from the manuscript (include quotes in quotation marks "like this" to indicate direct quotes from your manuscript), or elaborate on this item by providing additional information not in the ms, or briefly explain why the item is not applicable/relevant for your study

this wasnt applicable as no changes were implmented after trial commencement

3b-i) Bug fixes, Downtimes, Content Changes

Bug fixes, Downtimes, Content Changes: ehealth systems are often dynamic systems. A description of changes to methods therefore also includes important changes made on the intervention or comparator during the trial (e.g., major bug fixes or changes in the functionality or content) (5-iii) and other "unexpected events" that may have influenced study design such as staff changes, system failures/downtimes, etc. [2].

	1	2	3	4	5	
subitem not at all important	○	◉	○	○	○	essential

Clear selection

You're editing your response. Sharing this URL allows others to also edit your response.

FILL IN A NEW RESPONSE

Does your paper address subitem 3b-i?

Copy and paste relevant sections from the manuscript (include quotes in quotation marks "like this" to indicate direct quotes from your manuscript), or elaborate on this item by providing additional information not in the ms, or briefly explain why the item is not applicable/relevant for your study

Protocol Changes
The following modifications were made to the protocol following pre-registration but prior to the pilot trial:
Intervention Adjustments: After testing with three volunteers prior to recruitment, we added an initial prompt to the mentalisation-based intervention to encourage self-mentalisation, following feedback from that participants struggled to engage in mentalising without first acknowledging their own internal responses.
Participation Incentives: Lowered the minimum participation rate required for payment from 70% to 60% to increase feedback capture via the post-intervention questionnaire.

4a) Eligibility criteria for participants

Does your paper address CONSORT subitem 4a? *

Copy and paste relevant sections from the manuscript (include quotes in quotation marks "like this" to indicate direct quotes from your manuscript), or elaborate on this item by providing additional information not in the ms, or briefly explain why the item is not applicable/relevant for your study

Eligibility Criteria
Inclusion criteria: aged 18 years or above, currently experiencing symptoms of anxiety and/or depression (scoring in clinical range on either the PHQ-9, i.e. >9 or GAD-7, i.e. >7), able to access and use a personal smartphone, had daily contact with a partner, family member(s) or friend, were able to read and write in English and were registered with a GP in the UK.
Exclusion criteria: currently receiving psychological therapy, taking medication for their mental health or expressing any suicidal ideation or thoughts of self-harm at the point of screening (i.e. scored >0 on question nine of the PHQ-9).

You're editing your response. Sharing this URL allows others to also edit your response.

FILL IN A NEW RESPONSE

4a-i) Computer / Internet literacy

Computer / Internet literacy is often an implicit “de facto” eligibility criterion - this should be explicitly clarified.

	1	2	3	4	5	
subitem not at all important	○	○	○	○	◉	essential

Clear selection

Does your paper address subitem 4a-i?

Copy and paste relevant sections from the manuscript (include quotes in quotation marks "like this" to indicate direct quotes from your manuscript), or elaborate on this item by providing additional information not in the ms, or briefly explain why the item is not applicable/relevant for your study

"able to access and use a personal smartphone"

4a-ii) Open vs. closed, web-based vs. face-to-face assessments:

Open vs. closed, web-based vs. face-to-face assessments: Mention how participants were recruited (online vs. offline), e.g., from an open access website or from a clinic, and clarify if this was a purely web-based trial, or there were face-to-face components (as part of the intervention or for assessment), i.e., to what degree got the study team to know the participant. In online-only trials, clarify if participants were quasi-anonymous and whether having multiple identities was possible or whether technical or logistical measures (e.g., cookies, email confirmation, phone calls) were used to detect/prevent these.

	1	2	3	4	5	
subitem not at all important	○	○	○	○	◉	essential

Clear selection

You're editing your response. Sharing this URL allows others to also edit your response.

FILL IN A NEW RESPONSE

Does your paper address subitem 4a-ii? *

Copy and paste relevant sections from the manuscript (include quotes in quotation marks "like this" to indicate direct quotes from your manuscript), or elaborate on this item by providing additional information not in the ms, or briefly explain why the item is not applicable/relevant for your study

"Participants accessed study information, consent forms, and screening questionnaires online." "EMA Protocol: All participants completed identical ecological momentary assessments four times daily for 28 days, scheduled around individual sleep-wake times." "Participants were quasi-anonymous, personal identifibable information was collected at screening (and stored separately and securely) and a participant ID allocated thereafter. "

4a-iii) Information giving during recruitment

Information given during recruitment. Specify how participants were briefed for recruitment and in the informed consent procedures (e.g., publish the informed consent documentation as appendix, see also item X26), as this information may have an effect on user self-selection, user expectation and may also bias results.

	1	2	3	4	5	
subitem not at all important	○	○	○	◉	○	essential

Clear selection

Does your paper address subitem 4a-iii?

Copy and paste relevant sections from the manuscript (include quotes in quotation marks "like this" to indicate direct quotes from your manuscript), or elaborate on this item by providing additional information not in the ms, or briefly explain why the item is not applicable/relevant for your study

All participants were provided with details of the study, confirmed eligibility, and provided informed written consent (see Appendix for informed consent documentation).

4b) Settings and locations where the data were collected

You're editing your response. Sharing this URL allows others to also edit your response.

FILL IN A NEW RESPONSE

Does your paper address CONSORT subitem 4b? *

Copy and paste relevant sections from the manuscript (include quotes in quotation marks "like this" to indicate direct quotes from your manuscript), or elaborate on this item by providing additional information not in the ms, or briefly explain why the item is not applicable/relevant for your study

"Participants were recruited via convenience sampling through the University. "

4b-i) Report if outcomes were (self-)assessed through online questionnaires

Clearly report if outcomes were (self-)assessed through online questionnaires (as common in web-based trials) or otherwise.

	1	2	3	4	5	
subitem not at all important	○	○	○	○	◉	essential

Clear selection

Does your paper address subitem 4b-i? *

Copy and paste relevant sections from the manuscript (include quotes in quotation marks "like this" to indicate direct quotes from your manuscript), or elaborate on this item by providing additional information not in the ms, or briefly explain why the item is not applicable/relevant for your study

"Participants accessed study information, consent forms, and screening questionnaires online."

You're editing your response. Sharing this URL allows others to also edit your response.

FILL IN A NEW RESPONSE

4b-ii) Report how institutional affiliations are displayed

Report how institutional affiliations are displayed to potential participants [on ehealth media], as affiliations with prestigious hospitals or universities may affect volunteer rates, use, and reactions with regards to an intervention.(Not a required item – describe only if this may bias results)

	1	2	3	4	5	
subitem not at all important	◉	○	○	○	○	essential

Clear selection

Does your paper address subitem 4b-ii?

Copy and paste relevant sections from the manuscript (include quotes in quotation marks "like this" to indicate direct quotes from your manuscript), or elaborate on this item by providing additional information not in the ms, or briefly explain why the item is not applicable/relevant for your study

This is not applicable. This is not applicable.

5) The interventions for each group with sufficient details to allow replication, including how and when they were actually administered

5-i) Mention names, credential, affiliations of the developers, sponsors, and owners

Mention names, credential, affiliations of the developers, sponsors, and owners [6] (if authors/evaluators are owners or developer of the software, this needs to be declared in a “Conflict of interest” section or mentioned elsewhere in the manuscript).

	1	2	3	4	5	
subitem not at all important	◉	○	○	○	○	essential

Clear selection

You're editing your response. Sharing this URL allows others to also edit your response.

FILL IN A NEW RESPONSE

Does your paper address subitem 5-i?

Copy and paste relevant sections from the manuscript (include quotes in quotation marks "like this" to indicate direct quotes from your manuscript), or elaborate on this item by providing additional information not in the ms, or briefly explain why the item is not applicable/relevant for your study

its not a commercial product so not a COI

5-ii) Describe the history/development process

Describe the history/development process of the application and previous formative evaluations (e.g., focus groups, usability testing), as these will have an impact on adoption/use rates and help with interpreting results.

	1	2	3	4	5	
subitem not at all important	○	○	○	○	◉	essential

Clear selection

Does your paper address subitem 5-ii?

Copy and paste relevant sections from the manuscript (include quotes in quotation marks "like this" to indicate direct quotes from your manuscript), or elaborate on this item by providing additional information not in the ms, or briefly explain why the item is not applicable/relevant for your study

"Intervention Adjustments: After testing with three volunteers prior to recruitment, we added an initial prompt to the mentalisation-based intervention to encourage self-mentalisation, following feedback from that participants struggled to engage in mentalising without first acknowledging their own internal responses."

You're editing your response. Sharing this URL allows others to also edit your response.

FILL IN A NEW RESPONSE

5-iii) Revisions and updating

Revisions and updating. Clearly mention the date and/or version number of the application/intervention (and comparator, if applicable) evaluated, or describe whether the intervention underwent major changes during the evaluation process, or whether the development and/or content was “frozen” during the trial. Describe dynamic components such as news feeds or changing content which may have an impact on the replicability of the intervention (for unexpected events see item 3b).

	1	2	3	4	5	
subitem not at all important	○	○	●	○	○	essential

Clear selection

Does your paper address subitem 5-iii?

Copy and paste relevant sections from the manuscript (include quotes in quotation marks "like this" to indicate direct quotes from your manuscript), or elaborate on this item by providing additional information not in the ms, or briefly explain why the item is not applicable/relevant for your study

"The development and content were finalised and remained unchanged throughout the trial."

5-iv) Quality assurance methods

Provide information on quality assurance methods to ensure accuracy and quality of information provided [1], if applicable.

	1	2	3	4	5	
subitem not at all important	○	○	○	○	○	essential

You're editing your response. Sharing this URL allows others to also edit your response.

FILL IN A NEW RESPONSE

Does your paper address subitem 5-iv?

Copy and paste relevant sections from the manuscript (include quotes in quotation marks "like this" to indicate direct quotes from your manuscript), or elaborate on this item by providing additional information not in the ms, or briefly explain why the item is not applicable/relevant for your study

Feasibility Outcomes
We assessed feasibility through the following outcomes:
Recruitment rate was evaluated by tracking the number of individuals screened, the number meeting eligibility criteria, and the percentage of eligible participants who subsequently consented and were randomized to a study arm.
Retention rates was defined as the percentage of randomized participants who completed the final study assessment at [specify time point, e.g., end of intervention, 4-week follow-up]. Data was systematically collected on the number of participants at each stage of the study pipeline, from initial screening through to final data collection
Adherence rates were operationalized as the percentage of participants who engaged with at least 60% of the EMAs over the 4-week intervention period.
Engagement with the intervention content was measured by the average number of mindfulness or mentalization exercises completed per participant throughout the intervention period, as recorded by the application. These metrics allowed us to gauge participants' sustained interaction with both the assessment components and the therapeutic elements of the EMI.
Acceptability Outcomes
We assessed acceptability post intervention through a dedicated post-intervention questionnaire. This assessment aimed to understand participants' perceptions of the intervention's usability, utility, and overall experience. In addition to quantitative ratings, participants were provided with open-ended questions to offer qualitative feedback on their overall experience, including perceived benefits, challenges, suggestions for improvement, and any other relevant comments regarding the intervention and the EMI delivery method.

5-v) Ensure replicability by publishing the source code, and/or providing screenshots/screen-capture video, and/or providing flowcharts of the algorithms used

Ensure replicability by publishing the source code, and/or providing screenshots/screen-capture video, and/or providing flowcharts of the algorithms used. Replicability (i.e., other researchers should in principle be able to replicate the study) is a hallmark of scientific reporting.

	1	2	3	4	5	
subitem not at all important	○	○	○	○	◉	essential

You're editing your response. Sharing this URL allows others to also edit your response.

FILL IN A NEW RESPONSE

Does your paper address subitem 5-v?

Copy and paste relevant sections from the manuscript (include quotes in quotation marks "like this" to indicate direct quotes from your manuscript), or elaborate on this item by providing additional information not in the ms, or briefly explain why the item is not applicable/relevant for your study

"data and code are openly available" [OSF link]

5-vi) Digital preservation

Digital preservation: Provide the URL of the application, but as the intervention is likely to change or disappear over the course of the years; also make sure the intervention is archived (Internet Archive, webcitation.org, and/or publishing the source code or screenshots/videos alongside the article). As pages behind login screens cannot be archived, consider creating demo pages which are accessible without login.

	1	2	3	4	5	
subitem not at all important	◉	○	○	○	○	essential

Clear selection

Does your paper address subitem 5-vi?

Copy and paste relevant sections from the manuscript (include quotes in quotation marks "like this" to indicate direct quotes from your manuscript), or elaborate on this item by providing additional information not in the ms, or briefly explain why the item is not applicable/relevant for your study

this is not applicable as it was not a website

You're editing your response. Sharing this URL allows others to also edit your response.

FILL IN A NEW RESPONSE

5-vii) Access

Access: Describe how participants accessed the application, in what setting/context, if they had to pay (or were paid) or not, whether they had to be a member of specific group. If known, describe how participants obtained "access to the platform and Internet" [1]. To ensure access for editors/reviewers/readers, consider to provide a "backdoor" login account or demo mode for reviewers/readers to explore the application (also important for archiving purposes, see vi).

	1	2	3	4	5	
subitem not at all important	○	○	○	◉	○	essential

Clear selection

Does your paper address subitem 5-vii? *

Copy and paste relevant sections from the manuscript (include quotes in quotation marks "like this" to indicate direct quotes from your manuscript), or elaborate on this item by providing additional information not in the ms, or briefly explain why the item is not applicable/relevant for your study

" All participants downloaded the m-path app" "All participants (including those who chose to withdraw partway through) were entered into a prize draw to win a £50 (US $64) voucher. They received £20 (US $25) if they reached 60% completion of the EMA at the end of the study."

You're editing your response. Sharing this URL allows others to also edit your response.

FILL IN A NEW RESPONSE

5-viii) Mode of delivery, features/functionalities/components of the intervention and comparator, and the theoretical framework

Describe mode of delivery, features/functionalities/components of the intervention and comparator, and the theoretical framework [6] used to design them (instructional strategy [1], behaviour change techniques, persuasive features, etc., see e.g., [7, 8] for terminology). This includes an in-depth description of the content (including where it is coming from and who developed it) [1]," whether [and how] it is tailored to individual circumstances and allows users to track their progress and receive feedback" [6]. This also includes a description of communication delivery channels and – if computer-mediated communication is a component – whether communication was synchronous or asynchronous [6]. It also includes information on presentation strategies [1], including page design principles, average amount of text on pages, presence of hyperlinks to other resources, etc. [1].

	1	2	3	4	5	
subitem not at all important	○	○	○	○	◉	essential

Clear selection

You're editing your response. Sharing this URL allows others to also edit your response.

FILL IN A NEW RESPONSE

Does your paper address subitem 5-viii? *

Copy and paste relevant sections from the manuscript (include quotes in quotation marks "like this" to indicate direct quotes from your manuscript), or elaborate on this item by providing additional information not in the ms, or briefly explain why the item is not applicable/relevant for your study

Interventions
EMA Protocol: All participants downloaded the m-path app. They then completed identical ecological momentary assessments four times daily for 28 days, scheduled around individual sleep-wake times. Each assessment measured mood, stress, perceived expressed emotion (criticism, hostility, overinvolvement), warmth, and interpersonal behaviours on 7-point scales. Participants received video training on app use. Engagement was monitored, with email reminders sent when completion rates fell below 80% (maximum once weekly).
EMI phase began after 20 timepoints of baseline EMA, continuing for 21 days. The EMI was activated when any EE item ratings (criticism, hostility, overinvolvement or support) deviated >1 standard deviation from their rolling average. Interventions were delivered automatically via the mPath app.
Mentalisation-EMI arm: This micro-intervnetion was a structured reflection exercise on self and others' mental states with free-text response option. This was developed in line with the interpersonal affective focus, a repetitive pattern of relating to self and others (28). Teh first step encouraged self-focused mentalization, asking the client to reflect on their own perspective and understanding of a situation. Then encouraging other-focused mentalization, specifically asking the client to imagine the mental states (drives, motivations) and the intentions behind the other person's actions. The final step encouraged self-focused mentalization with a future orientation, asking the client to reflect on alternative responses and their own intentions for future behaviour.
Mindfulness arm: This micro intervention employed a three-step mindfulness breathing exercise. The first step encouraged internal awareness. Then promoting present-moment awareness and acceptance of one's current mental state, with an emphasis on physical sensations. The final step encouraged integration of multiple physical aspects of self-experience (whole body awareness). This could facilitate the connection between internal experience and how one might be perceived by others.

You're editing your response. Sharing this URL allows others to also edit your response.

FILL IN A NEW RESPONSE

5-ix) Describe use parameters

Describe use parameters (e.g., intended “doses” and optimal timing for use). Clarify what instructions or recommendations were given to the user, e.g., regarding timing, frequency, heaviness of use, if any, or was the intervention used ad libitum.

	1	2	3	4	5	
subitem not at all important	○	○	○	○	◉	essential

Clear selection

Does your paper address subitem 5-ix?

Copy and paste relevant sections from the manuscript (include quotes in quotation marks "like this" to indicate direct quotes from your manuscript), or elaborate on this item by providing additional information not in the ms, or briefly explain why the item is not applicable/relevant for your study

"They then completed identical ecological momentary assessments four times daily for 28 days, scheduled around individual sleep-wake times." "EMI phase began after 20 timepoints of baseline EMA, continuing for 21 days. The EMI was activated when any EE item ratings (criticism, hostility, overinvolvement or support) deviated >1 standard deviation from their rolling average. Interventions were delivered automatically via the mPath app."

5-x) Clarify the level of human involvement

Clarify the level of human involvement (care providers or health professionals, also technical assistance) in the e-intervention or as co-intervention (detail number and expertise of professionals involved, if any, as well as “type of assistance offered, the timing and frequency of the support, how it is initiated, and the medium by which the assistance is delivered”. It may be necessary to distinguish between the level of human involvement required for the trial, and the level of human involvement required for a routine application outside of a RCT setting (discuss under item 21 – generalizability).

	1	2	3	4	5	
subitem not at all important	○	○	○	○	◉	essential

Clear selection

You're editing your response. Sharing this URL allows others to also edit your response.

FILL IN A NEW RESPONSE

Does your paper address subitem 5-x?

Copy and paste relevant sections from the manuscript (include quotes in quotation marks "like this" to indicate direct quotes from your manuscript), or elaborate on this item by providing additional information not in the ms, or briefly explain why the item is not applicable/relevant for your study

this was fully automated - as outlined in the abstract.

5-xi) Report any prompts/reminders used

Report any prompts/reminders used: Clarify if there were prompts (letters, emails, phone calls, SMS) to use the application, what triggered them, frequency etc. It may be necessary to distinguish between the level of prompts/reminders required for the trial, and the level of prompts/reminders for a routine application outside of a RCT setting (discuss under item 21 – generalizability).

	1	2	3	4	5	
subitem not at all important	○	○	○	◉	○	essential

Clear selection

Does your paper address subitem 5-xi? *

Copy and paste relevant sections from the manuscript (include quotes in quotation marks "like this" to indicate direct quotes from your manuscript), or elaborate on this item by providing additional information not in the ms, or briefly explain why the item is not applicable/relevant for your study

" Engagement was monitored, with email reminders sent when completion rates fell below 80% (maximum once weekly)."

You're editing your response. Sharing this URL allows others to also edit your response.

FILL IN A NEW RESPONSE

5-xii) Describe any co-interventions (incl. training/support)

Describe any co-interventions (incl. training/support): Clearly state any interventions that are provided in addition to the targeted eHealth intervention, as ehealth intervention may not be designed as stand-alone intervention. This includes training sessions and support [1]. It may be necessary to distinguish between the level of training required for the trial, and the level of training for a routine application outside of a RCT setting (discuss under item 21 – generalizability.

	1	2	3	4	5	
subitem not at all important	◉	○	○	○	○	essential

Clear selection

Does your paper address subitem 5-xii? *

Copy and paste relevant sections from the manuscript (include quotes in quotation marks "like this" to indicate direct quotes from your manuscript), or elaborate on this item by providing additional information not in the ms, or briefly explain why the item is not applicable/relevant for your study

not applicable it was a stand alone intevention

6a) Completely defined pre-specified primary and secondary outcome measures, including how and when they were assessed

You're editing your response. Sharing this URL allows others to also edit your response.

FILL IN A NEW RESPONSE

Does your paper address CONSORT subitem 6a? *

Copy and paste relevant sections from the manuscript (include quotes in quotation marks "like this" to indicate direct quotes from your manuscript), or elaborate on this item by providing additional information not in the ms, or briefly explain why the item is not applicable/relevant for your study

You're editing your response. Sharing this URL allows others to also edit your response.

FILL IN A NEW RESPONSE

Feasibility Outcomes
We assessed feasibility through the following outcomes:
Recruitment rate was evaluated by tracking the number of individuals screened, the number meeting eligibility criteria, and the percentage of eligible participants who subsequently consented and were randomized to a study arm.
Retention rates was defined as the percentage of randomized participants who completed the final study assessment at [specify time point, e.g., end of intervention, 4-week follow-up]. Data was systematically collected on the number of participants at each stage of the study pipeline, from initial screening through to final data collection
Adherence rates were operationalized as the percentage of participants who engaged with at least 60% of the EMAs over the 4-week intervention period.
Engagement with the intervention content was measured by the average number of mindfulness or mentalization exercises completed per participant throughout the intervention period, as recorded by the application. These metrics allowed us to gauge participants' sustained interaction with both the assessment components and the therapeutic elements of the EMI.
Acceptability Outcomes
We assessed acceptability post intervention through a dedicated post-intervention questionnaire. This assessment aimed to understand participants' perceptions of the intervention's usability, utility, and overall experience. In addition to quantitative ratings, participants were provided with open-ended questions to offer qualitative feedback on their overall experience, including perceived benefits, challenges, suggestions for improvement, and any other relevant comments regarding the intervention and the EMI delivery method.
Potential Effectiveness Outcomes
We collected the following preliminary outcome data for a future definitive trial:
Primary outcomes:
Common mental health problems were assessed pre and post intervention using the PHQ-9, to assess depression (30) and the GAD-7, to assess generalised anxiety (31). Internal consistency within the sample was $\alpha = .72$ for each measure.
Ecological Momentary Assessment Measures: Participants completed ecological momentary assessments (EMA) throughout the day via smartphone notifications. Each assessment consisted of 10 single-item measures designed to capture momentary interpersonal experiences, emotional states, and behavioural responses. All items were rated on a scale of 0 to 10, with higher scores indicating greater intensity of the assessed construct. The prompt for each item began with "Since the last beep..." to capture experiences between assessment points. Items assessed interpersonal experiences (criticism, hostility, overinvolvement, support, and warmth), emotional states (mood and stress), behavioural responses (avoidance), and interpersonal stance (warmth/hostility and assertiveness). Specifically, participants rated their agreement with statements such as "Since the last beep I have felt criticized" (criticism) and "I feel valued or close to others" (warmth). For mood, participants responded to "How do you rate your mood generally?" with higher scores indicating more positive mood. For interpersonal stance, participants rated their reactions along a continuum from "Cold/Hostile" to "Warm/Friendly."

You're editing your response. Sharing this URL allows others to also edit your response.

FILL IN A NEW RESPONSE

6a-i) Online questionnaires: describe if they were validated for online use and apply CHERRIES items to describe how the questionnaires were designed/deployed

If outcomes were obtained through online questionnaires, describe if they were validated for online use and apply CHERRIES items to describe how the questionnaires were designed/deployed [9].

	1	2	3	4	5	
subitem not at all important	◉	○	○	○	○	essential

Clear selection

Does your paper address subitem 6a-i?

Copy and paste relevant sections from manuscript text

Your answer

6a-ii) Describe whether and how “use” (including intensity of use/dosage) was defined/measured/monitored

Describe whether and how “use” (including intensity of use/dosage) was defined/measured/monitored (logins, logfile analysis, etc.). Use/adoption metrics are important process outcomes that should be reported in any ehealth trial.

	1	2	3	4	5	
subitem not at all important	○	○	○	◉	○	essential

Clear selection

You're editing your response. Sharing this URL allows others to also edit your response.

FILL IN A NEW RESPONSE

Does your paper address subitem 6a-ii?

Copy and paste relevant sections from manuscript text

Adherence rates were operationalized as the percentage of participants who engaged with at least 60% of the EMAs over the 4-week intervention period.
Engagement with the intervention content was measured by the average number of mindfulness or mentalization exercises completed per participant throughout the intervention period, as recorded by the application. These metrics allowed us to gauge participants' sustained interaction with both the assessment components and the therapeutic elements of the EMI.

6a-iii) Describe whether, how, and when qualitative feedback from participants was obtained

Describe whether, how, and when qualitative feedback from participants was obtained (e.g., through emails, feedback forms, interviews, focus groups).

	1	2	3	4	5	
subitem not at all important	○	○	○	◉	○	essential

Clear selection

Does your paper address subitem 6a-iii?

Copy and paste relevant sections from manuscript text

Acceptability Outcomes
We assessed acceptability post intervention through a dedicated post-intervention questionnaire. This assessment aimed to understand participants' perceptions of the intervention's usability, utility, and overall experience. In addition to quantitative ratings, participants were provided with open-ended questions to offer qualitative feedback on their overall experience, including perceived benefits, challenges, suggestions for improvement, and any other relevant comments regarding the intervention and the EMI delivery method.

6b) Any changes to trial outcomes after the trial commenced, with reasons

You're editing your response. Sharing this URL allows others to also edit your response.

FILL IN A NEW RESPONSE

Does your paper address CONSORT subitem 6b? *

Copy and paste relevant sections from the manuscript (include quotes in quotation marks "like this" to indicate direct quotes from your manuscript), or elaborate on this item by providing additional information not in the ms, or briefly explain why the item is not applicable/relevant for your study

not applicable no changes

7a) How sample size was determined

NPT: When applicable, details of whether and how the clustering by care provides or centers was addressed

7a-i) Describe whether and how expected attrition was taken into account when calculating the sample size

Describe whether and how expected attrition was taken into account when calculating the sample size.

	1	2	3	4	5	
subitem not at all important	○	○	○	◉	○	essential

Clear selection

You're editing your response. Sharing this URL allows others to also edit your response.

FILL IN A NEW RESPONSE

Does your paper address subitem 7a-i?

Copy and paste relevant sections from manuscript title (include quotes in quotation marks "like this" to indicate direct quotes from your manuscript), or elaborate on this item by providing additional information not in the ms, or briefly explain why the item is not applicable/relevant for your study

Sample Size
We determined a sample size of 80 participants (40 in each group) based on methodological considerations for feasibility studies involving EMA (34). The sample size calculation considered the practical constraints of managing intensive data collection (approximately 4 assessments per day for 28 days, yielding 112 potential observations per participant) while ensuring adequate representation to estimate parameters for the main study. With an anticipated 70% completion rate of EMA prompts, we expect approximately 6,272 completed assessments across all participants, providing sufficient statistical power to conduct preliminary analyses of temporal patterns and associations between measured variables. Our sample size provided robust data to estimate between-group differences, within-person variability (35). This sample size is also sufficient to estimate compliance rates, adherence patterns, and variability in EMA responses, test the usability of the EMA protocol, and detect technical issues that might affect implementation in a larger trial (36,37).

7b) When applicable, explanation of any interim analyses and stopping guidelines

Does your paper address CONSORT subitem 7b? *

Copy and paste relevant sections from the manuscript (include quotes in quotation marks "like this" to indicate direct quotes from your manuscript), or elaborate on this item by providing additional information not in the ms, or briefly explain why the item is not applicable/relevant for your study

this is not applicable to the study

8a) Method used to generate the random allocation sequence
NPT: When applicable, how care providers were allocated to each trial group

You're editing your response. Sharing this URL allows others to also edit your response.

FILL IN A NEW RESPONSE

Does your paper address CONSORT subitem 8a? *

Copy and paste relevant sections from the manuscript (include quotes in quotation marks "like this" to indicate direct quotes from your manuscript), or elaborate on this item by providing additional information not in the ms, or briefly explain why the item is not applicable/relevant for your study

"This pilot randomized controlled trial used a parallel-group design with a 1:1 allocation ratio. " "COD generated the random allocation sequence, while SOR and AJ enrolled participants, and assigned participants to interventions."

8b) Type of randomisation; details of any restriction (such as blocking and block size)

Does your paper address CONSORT subitem 8b? *

Copy and paste relevant sections from the manuscript (include quotes in quotation marks "like this" to indicate direct quotes from your manuscript), or elaborate on this item by providing additional information not in the ms, or briefly explain why the item is not applicable/relevant for your study

random allocation sequence with a 1:1 allocation ratio

9) Mechanism used to implement the random allocation sequence (such as sequentially numbered containers), describing any steps taken to conceal the sequence until interventions were assigned

Does your paper address CONSORT subitem 9? *

Copy and paste relevant sections from the manuscript (include quotes in quotation marks "like this" to indicate direct quotes from your manuscript), or elaborate on this item by providing additional information not in the ms, or briefly explain why the item is not applicable/relevant for your study

COD generated the random allocation sequence, while SOR and AJ enrolled participants, and assigned participants to interventions."

You're editing your response. Sharing this URL allows others to also edit your response.

FILL IN A NEW RESPONSE

10) Who generated the random allocation sequence, who enrolled participants, and who assigned participants to interventions

Does your paper address CONSORT subitem 10? *

Copy and paste relevant sections from the manuscript (include quotes in quotation marks "like this" to indicate direct quotes from your manuscript), or elaborate on this item by providing additional information not in the ms, or briefly explain why the item is not applicable/relevant for your study

"COD generated the random allocation sequence, while SOR and AJ enrolled participants, and assigned participants to interventions."

11a) If done, who was blinded after assignment to interventions (for example, participants, care providers, those assessing outcomes) and how

NPT: Whether or not administering co-interventions were blinded to group assignment

11a-i) Specify who was blinded, and who wasn't

Specify who was blinded, and who wasn't. Usually, in web-based trials it is not possible to blind the participants [1, 3] (this should be clearly acknowledged), but it may be possible to blind outcome assessors, those doing data analysis or those administering co-interventions (if any).

	1	2	3	4	5	
subitem not at all important	○	○	○	◉	○	essential

Clear selection

You're editing your response. Sharing this URL allows others to also edit your response.

FILL IN A NEW RESPONSE

Does your paper address subitem 11a-i? *

Copy and paste relevant sections from the manuscript (include quotes in quotation marks "like this" to indicate direct quotes from your manuscript), or elaborate on this item by providing additional information not in the ms, or briefly explain why the item is not applicable/relevant for your study

"Participants were blind to group allocation and of the theoretical basis of the intervention. Allocation was concealed through the app's automated system. Researchers were not blind to group allocation due to practicalities of administering the study. "

11a-ii) Discuss e.g., whether participants knew which intervention was the "intervention of interest" and which one was the "comparator"

Informed consent procedures (4a-ii) can create biases and certain expectations - discuss e.g., whether participants knew which intervention was the "intervention of interest" and which one was the "comparator".

	1	2	3	4	5	
subitem not at all important	○	○	○	◉	○	essential

Clear selection

Does your paper address subitem 11a-ii?

Copy and paste relevant sections from the manuscript (include quotes in quotation marks "like this" to indicate direct quotes from your manuscript), or elaborate on this item by providing additional information not in the ms, or briefly explain why the item is not applicable/relevant for your study

this is not applicable as both arms were of interest.

11b) If relevant, description of the similarity of interventions

(this item is usually not relevant for ehealth trials as it refers to similarity of a placebo or sham intervention to a active medication/intervention)

You're editing your response. Sharing this URL allows others to also edit your response.

FILL IN A NEW RESPONSE

Does your paper address CONSORT subitem 11b? *

Copy and paste relevant sections from the manuscript (include quotes in quotation marks "like this" to indicate direct quotes from your manuscript), or elaborate on this item by providing additional information not in the ms, or briefly explain why the item is not applicable/relevant for your study

"Mentalisation-EMI arm: This micro-intervnetion was a structured reflection exercise on self and others' mental states with free-text response option. This was developed in line with the interpersonal affective focus, a repetitive pattern of relating to self and others (28). Teh first step encouraged self-focused mentalization, asking the client to reflect on their own perspective and understanding of a situation. Then encouraging other-focused mentalization, specifically asking the client to imagine the mental states (drives, motivations) and the intentions behind the other person's actions. The final step encouraged self-focused mentalization with a future orientation, asking the client to reflect on alternative responses and their own intentions for future behaviour.
Mindfulness arm: This micro intervention employed a three-step mindfulness breathing exercise. The first step encouraged internal awareness. Then promoting present-moment awareness and acceptance of one's current mental state, with an emphasis on physical sensations. The final step encouraged integration of multiple physical aspects of self-experience (whole body awareness). This could facilitate the connection between internal experience and how one might be perceived by others.
"

12a) Statistical methods used to compare groups for primary and secondary outcomes

NPT: When applicable, details of whether and how the clustering by care providers or centers was addressed

You're editing your response. Sharing this URL allows others to also edit your response.

FILL IN A NEW RESPONSE

Does your paper address CONSORT subitem 12a? *

Copy and paste relevant sections from the manuscript (include quotes in quotation marks "like this" to indicate direct quotes from your manuscript), or elaborate on this item by providing additional information not in the ms, or briefly explain why the item is not applicable/relevant for your study

"Analysis of pre/post Outcomes
We conducted between-group and within-group analyses to evaluate the effects of the interventions. For between-group comparisons, we used Analysis of Covariance (ANCOVA) with post-intervention scores as dependent variables, intervention group (Mentalization vs. Mindfulness) as the independent variable, and pre-intervention scores as covariates. This approach accounts for baseline differences and provides adjusted post-intervention means for each group.
For within-group analyses, we conducted paired t-tests to evaluate pre-post changes within each intervention arm. Cohen's d was calculated to quantify the magnitude of change, using the standard deviation of the difference scores as the denominator, as recommended for paired designs.
Qualitative data in the participant feedback survey was analysed and key themes were identified and summarised quantitatively using inductive quantitative content analysis, a technique entailing the systematic quantitative analysis of message characteristics in text (38).
EMI analysis
To analyze the EMA data, we employed a linear mixed-effects modeling approach that accounted for the hierarchical structure of repeated measurements nested within participants (31). For each EMA item, we fitted the following model: $outcome\_ij = \beta_0 + \beta_1(treatment) + \beta_2(group) + \beta_3(treatment \times group) + u_{0i} + \varepsilon\_ij$ where outcome_ij represents the EMA item for participant i at measurement occasion j, β_0 is the overall intercept, β_1 represents the main effect of treatment condition (i.e. baseline/intervention phase), β_2 represents the main effect of group (mentalization/mindfulness), and β_3 represents the interaction between treatment and group. The model included a random intercept for each participant (u_{0i}) to account for between-person variability in baseline levels of the outcome. To address the temporal dependency in the repeated measurements, we specified a first-order autoregressive (AR1) correlation structure with a 0.01 initial correlation value. The models were estimated using restricted maximum likelihood (REML).
To quantify intervention effects, we calculated standardized mean difference effect sizes. We calculated both within-group effect sizes to evaluate baseline-intervention phase changes for each group separately and between-group effect sizes to compare treatment effects across groups. For within-group effect sizes, we calculated how much the treatment affected each group individually. For between-group comparisons, we calculated the differences in treatment effects when comparing one group to another.
All analyses were conducted in R (39) version 4.0.3, using the `nlme` package (40) for model fitting and the `scdhlm` package (41) for effect size calculations."

You're editing your response. Sharing this URL allows others to also edit your response.

FILL IN A NEW RESPONSE

12a-i) Imputation techniques to deal with attrition / missing values

Imputation techniques to deal with attrition / missing values: Not all participants will use the intervention/comparator as intended and attrition is typically high in ehealth trials. Specify how participants who did not use the application or dropped out from the trial were treated in the statistical analysis (a complete case analysis is strongly discouraged, and simple imputation techniques such as LOCF may also be problematic [4]).

	1	2	3	4	5	
subitem not at all important	(●)	○	○	○	○	essential

Clear selection

Does your paper address subitem 12a-i? *

Copy and paste relevant sections from the manuscript (include quotes in quotation marks "like this" to indicate direct quotes from your manuscript), or elaborate on this item by providing additional information not in the ms, or briefly explain why the item is not applicable/relevant for your study

not applicable - no imputation

12b) Methods for additional analyses, such as subgroup analyses and adjusted analyses

Does your paper address CONSORT subitem 12b? *

Copy and paste relevant sections from the manuscript (include quotes in quotation marks "like this" to indicate direct quotes from your manuscript), or elaborate on this item by providing additional information not in the ms, or briefly explain why the item is not applicable/relevant for your study

not applicable no subgroup analyses

X26) REB/IRB Approval and Ethical Considerations [recommended as subheading

You're editing your response. Sharing this URL allows others to also edit your response.

FILL IN A NEW RESPONSE

X26-i) Comment on ethics committee approval

	1	2	3	4	5	
subitem not at all important	○	○	○	●	○	essential

Clear selection

Does your paper address subitem X26-i?

Copy and paste relevant sections from the manuscript (include quotes in quotation marks "like this" to indicate direct quotes from your manuscript), or elaborate on this item by providing additional information not in the ms, or briefly explain why the item is not applicable/relevant for your study

" REB/IRB Approval and Ethical Considerations
Ethical approval was granted by the University College London (UCL) Research Ethics Department (26261/00). "

x26-ii) Outline informed consent procedures

Outline informed consent procedures e.g., if consent was obtained offline or online (how? Checkbox, etc.?), and what information was provided (see 4a-ii). See [6] for some items to be included in informed consent documents.

	1	2	3	4	5	
subitem not at all important	○	○	○	●	○	essential

Clear selection

You're editing your response. Sharing this URL allows others to also edit your response.

FILL IN A NEW RESPONSE

Does your paper address subitem X26-ii?

Copy and paste relevant sections from the manuscript (include quotes in quotation marks "like this" to indicate direct quotes from your manuscript), or elaborate on this item by providing additional information not in the ms, or briefly explain why the item is not applicable/relevant for your study

"All participants were provided with details of the study, confirmed eligibility, and provided informed written consent (see Appendix for informed consent documentation). "

X26-iii) Safety and security procedures

Safety and security procedures, incl. privacy considerations, and any steps taken to reduce the likelihood or detection of harm (e.g., education and training, availability of a hotline)

	1	2	3	4	5	
subitem not at all important	○	○	○	◉	○	essential

Clear selection

Does your paper address subitem X26-iii?

Copy and paste relevant sections from the manuscript (include quotes in quotation marks "like this" to indicate direct quotes from your manuscript), or elaborate on this item by providing additional information not in the ms, or briefly explain why the item is not applicable/relevant for your study

"Risk Management Protocol
Risk assessment occurred at screening and study completion. Structured risk management protocols were implemented for participants reporting suicidal ideation, including signposting or telephone assessment by the research team.
"

RESULTS

You're editing your response. Sharing this URL allows others to also edit your response.

FILL IN A NEW RESPONSE

13a) For each group, the numbers of participants who were randomly assigned, received intended treatment, and were analysed for the primary outcome
NPT: The number of care providers or centers performing the intervention in each group and the number of patients treated by each care provider in each center

Does your paper address CONSORT subitem 13a? *

Copy and paste relevant sections from the manuscript (include quotes in quotation marks "like this" to indicate direct quotes from your manuscript), or elaborate on this item by providing additional information not in the ms, or briefly explain why the item is not applicable/relevant for your study

this is outlined in Figure 1: a CONSORT flow diagram showing participant flow through each stage of the pilot trial

13b) For each group, losses and exclusions after randomisation, together with reasons

Does your paper address CONSORT subitem 13b? (NOTE: Preferably, this is shown in a CONSORT flow diagram) *

Copy and paste relevant sections from the manuscript (include quotes in quotation marks "like this" to indicate direct quotes from your manuscript), or elaborate on this item by providing additional information not in the ms, or briefly explain why the item is not applicable/relevant for your study

this is outlined in Figure 1: a CONSORT flow diagram showing participant flow through each stage of the pilot trial

You're editing your response. Sharing this URL allows others to also edit your response.

FILL IN A NEW RESPONSE

13b-i) Attrition diagram

Strongly recommended: An attrition diagram (e.g., proportion of participants still logging in or using the intervention/comparator in each group plotted over time, similar to a survival curve) or other figures or tables demonstrating usage/dose/engagement.

	1	2	3	4	5	
subitem not at all important	○	○	○	◉	○	essential

Clear selection

Does your paper address subitem 13b-i?

Copy and paste relevant sections from the manuscript or cite the figure number if applicable (include quotes in quotation marks "like this" to indicate direct quotes from your manuscript), or elaborate on this item by providing additional information not in the ms, or briefly explain why the item is not applicable/relevant for your study

this is outlined in Figure 1: a CONSORT flow diagram showing participant flow through each stage of the pilot trial

14a) Dates defining the periods of recruitment and follow-up

Does your paper address CONSORT subitem 14a? *

Copy and paste relevant sections from the manuscript (include quotes in quotation marks "like this" to indicate direct quotes from your manuscript), or elaborate on this item by providing additional information not in the ms, or briefly explain why the item is not applicable/relevant for your study

Recruitment
Participants were recruited between March and December 2024, with final follow-up assessments completed by February 2025. The target recruitment rate of 10 participants per month was met overall, although recruitment slowed during the summer months due to seasonal variation.

You're editing your response. Sharing this URL allows others to also edit your response.

FILL IN A NEW RESPONSE

14a-i) Indicate if critical “secular events” fell into the study period

Indicate if critical “secular events” fell into the study period, e.g., significant changes in Internet resources available or “changes in computer hardware or Internet delivery resources”

	1	2	3	4	5	
subitem not at all important	◉	○	○	○	○	essential

Clear selection

Does your paper address subitem 14a-i?

Copy and paste relevant sections from the manuscript (include quotes in quotation marks "like this" to indicate direct quotes from your manuscript), or elaborate on this item by providing additional information not in the ms, or briefly explain why the item is not applicable/relevant for your study

this wasnt applicable to study

14b) Why the trial ended or was stopped (early)

Does your paper address CONSORT subitem 14b? *

Copy and paste relevant sections from the manuscript (include quotes in quotation marks "like this" to indicate direct quotes from your manuscript), or elaborate on this item by providing additional information not in the ms, or briefly explain why the item is not applicable/relevant for your study

Recruitment
Participants were recruited between March and December 2024, with final follow-up assessments completed by February 2025.

You're editing your response. Sharing this URL allows others to also edit your response.

FILL IN A NEW RESPONSE

15) A table showing baseline demographic and clinical characteristics for each group
NPT: When applicable, a description of care providers (case volume, qualification, expertise, etc.) and centers (volume) in each group

Does your paper address CONSORT subitem 15? *

Copy and paste relevant sections from the manuscript (include quotes in quotation marks "like this" to indicate direct quotes from your manuscript), or elaborate on this item by providing additional information not in the ms, or briefly explain why the item is not applicable/relevant for your study

Table 1 presents the baseline characteristics of participants by trial arm.

15-i) Report demographics associated with digital divide issues

In ehealth trials it is particularly important to report demographics associated with digital divide issues, such as age, education, gender, social-economic status, computer/Internet/ehealth literacy of the participants, if known.

	1	2	3	4	5	
subitem not at all important	○	○	○	●	○	essential

Clear selection

Does your paper address subitem 15-i? *

Copy and paste relevant sections from the manuscript (include quotes in quotation marks "like this" to indicate direct quotes from your manuscript), or elaborate on this item by providing additional information not in the ms, or briefly explain why the item is not applicable/relevant for your study

Table 1 presents the baseline characteristics of participants by trial arm.

You're editing your response. Sharing this URL allows others to also edit your response.

FILL IN A NEW RESPONSE

16) For each group, number of participants (denominator) included in each analysis and whether the analysis was by original assigned groups

16-i) Report multiple “denominators” and provide definitions

Report multiple “denominators” and provide definitions: Report N’s (and effect sizes) “across a range of study participation [and use] thresholds” [1], e.g., N exposed, N consented, N used more than x times, N used more than y weeks, N participants “used” the intervention/comparator at specific pre-defined time points of interest (in absolute and relative numbers per group). Always clearly define “use” of the intervention.

	1	2	3	4	5	
subitem not at all important	○	○	○	◉	○	essential

Clear selection

Does your paper address subitem 16-i? *

Copy and paste relevant sections from the manuscript (include quotes in quotation marks "like this" to indicate direct quotes from your manuscript), or elaborate on this item by providing additional information not in the ms, or briefly explain why the item is not applicable/relevant for your study

the number remained constant during analysis and is reported.

16-ii) Primary analysis should be intent-to-treat

Primary analysis should be intent-to-treat, secondary analyses could include comparing only “users”, with the appropriate caveats that this is no longer a randomized sample (see 18-i).

	1	2	3	4	5	
subitem not at all important	○	○	○	○	○	essential

You're editing your response. Sharing this URL allows others to also edit your response.

FILL IN A NEW RESPONSE

Does your paper address subitem 16-ii?

Copy and paste relevant sections from the manuscript (include quotes in quotation marks "like this" to indicate direct quotes from your manuscript), or elaborate on this item by providing additional information not in the ms, or briefly explain why the item is not applicable/relevant for your study

this is a feasability trial and all data are presented for users, with baseline demogrpahic comparisons. all stastical analysis are secondary to the acceptability and feasability findings.

17a) For each primary and secondary outcome, results for each group, and the estimated effect size and its precision (such as 95% confidence interval)

Does your paper address CONSORT subitem 17a? *

Copy and paste relevant sections from the manuscript (include quotes in quotation marks "like this" to indicate direct quotes from your manuscript), or elaborate on this item by providing additional information not in the ms, or briefly explain why the item is not applicable/relevant for your study

Both interventions showed significant improvements on several outcome measures. In the mentalization group, significant reductions were observed in anxiety symptoms (GAD-7: pre $M = 12.30$ (3.58); post $M = 8.19$ (4.25); $t = -4.94$, $p < .001$, $d = -0.81$, 95% CI [-1.15, -0.47]), and depression symptoms (PHQ-9: pre $M = 12.05$ (3.80); post $M = 7.87$ (4.29); $t = -4.24$, $p < .001$, $d = -0.70$, 95% CI [-1.03, -0.37]).
Similarly, the mindfulness group demonstrated significant reductions in anxiety (GAD-7: pre $M = 11.11$ (4.48); post $M = 7.45$ (4.30); $t = -4.16$, $p < .001$, $d = -0.68$, 95% CI [-1.01, -0.35]), depression (PHQ-9: pre $M = 11.53$ (4.40); post $M = 7.32$ (3.86); $t = -4.41$, $p < .001$, $d = -0.72$, 95% CI [-1.05, -0.39]).

You're editing your response. Sharing this URL allows others to also edit your response.

FILL IN A NEW RESPONSE

17a-i) Presentation of process outcomes such as metrics of use and intensity of use

In addition to primary/secondary (clinical) outcomes, the presentation of process outcomes such as metrics of use and intensity of use (dose, exposure) and their operational definitions is critical. This does not only refer to metrics of attrition (13-b) (often a binary variable), but also to more continuous exposure metrics such as “average session length”. These must be accompanied by a technical description how a metric like a “session” is defined (e.g., timeout after idle time) [1] (report under item 6a).

	1	2	3	4	5	
subitem not at all important	○	○	○	◉	○	essential

Clear selection

Does your paper address subitem 17a-i?

Copy and paste relevant sections from the manuscript (include quotes in quotation marks "like this" to indicate direct quotes from your manuscript), or elaborate on this item by providing additional information not in the ms, or briefly explain why the item is not applicable/relevant for your study

these are outlined in detail in the feasability and acceptability outcome in the result section (too large to copy in here)

17b) For binary outcomes, presentation of both absolute and relative effect sizes is recommended

Does your paper address CONSORT subitem 17b? *

Copy and paste relevant sections from the manuscript (include quotes in quotation marks "like this" to indicate direct quotes from your manuscript), or elaborate on this item by providing additional information not in the ms, or briefly explain why the item is not applicable/relevant for your study

this was not applicable to the data

You're editing your response. Sharing this URL allows others to also edit your response.

FILL IN A NEW RESPONSE

18) Results of any other analyses performed, including subgroup analyses and adjusted analyses, distinguishing pre-specified from exploratory

Does your paper address CONSORT subitem 18? *

Copy and paste relevant sections from the manuscript (include quotes in quotation marks "like this" to indicate direct quotes from your manuscript), or elaborate on this item by providing additional information not in the ms, or briefly explain why the item is not applicable/relevant for your study

all analysis were pre registered https://aspredicted.org/kn7g-xkhg.pdf We also outline where exploratory analysis was conducted in the results section "Exploratory analysis investigated whether ... "

18-i) Subgroup analysis of comparing only users

A subgroup analysis of comparing only users is not uncommon in ehealth trials, but if done, it must be stressed that this is a self-selected sample and no longer an unbiased sample from a randomized trial (see 16-iii).

	1	2	3	4	5	
subitem not at all important	(•)	( )	( )	( )	( )	essential

Clear selection

Does your paper address subitem 18-i?

Copy and paste relevant sections from the manuscript (include quotes in quotation marks "like this" to indicate direct quotes from your manuscript), or elaborate on this item by providing additional information not in the ms, or briefly explain why the item is not applicable/relevant for your study

this was not relevant to this study.

19) All important harms or unintended effects in each group

[illegible]

You're editing your response. Sharing this URL allows others to also edit your response.

FILL IN A NEW RESPONSE

Does your paper address CONSORT subitem 19? *

Copy and paste relevant sections from the manuscript (include quotes in quotation marks "like this" to indicate direct quotes from your manuscript), or elaborate on this item by providing additional information not in the ms, or briefly explain why the item is not applicable/relevant for your study

"One participant in the mindfulness condition withdrew due to perceived negative effects on their mental health, specifically an increase in body image-related distress linked to the intervention's focus on bodily awareness. This was the only adverse reaction reported during the trial."

19-i) Include privacy breaches, technical problems

Include privacy breaches, technical problems. This does not only include physical "harm" to participants, but also incidents such as perceived or real privacy breaches [1], technical problems, and other unexpected/unintended incidents. "Unintended effects" also includes unintended positive effects [2].

	1	2	3	4	5	
subitem not at all important	(•)	( )	( )	( )	( )	essential

Clear selection

Does your paper address subitem 19-i?

Copy and paste relevant sections from the manuscript (include quotes in quotation marks "like this" to indicate direct quotes from your manuscript), or elaborate on this item by providing additional information not in the ms, or briefly explain why the item is not applicable/relevant for your study

this was not applicable to this study

You're editing your response. Sharing this URL allows others to also edit your response.

FILL IN A NEW RESPONSE

19-ii) Include qualitative feedback from participants or observations from staff/researchers

Include qualitative feedback from participants or observations from staff/researchers, if available, on strengths and shortcomings of the application, especially if they point to unintended/unexpected effects or uses. This includes (if available) reasons for why people did or did not use the application as intended by the developers.

	1	2	3	4	5	
subitem not at all important	○	○	○	●	○	essential

Clear selection

Does your paper address subitem 19-ii?

Copy and paste relevant sections from the manuscript (include quotes in quotation marks "like this" to indicate direct quotes from your manuscript), or elaborate on this item by providing additional information not in the ms, or briefly explain why the item is not applicable/relevant for your study

this was gathered and reported on "Participants' qualitative feedback (n=32, 42.67% of study completers) was thematically coded (see supplementary materials). The mentalization group was significantly more likely to provide qualitative feedback than the mindfulness group (21 vs. 11 participants; $\chi^2(1) = 5.927$, $p = .015$).
Participants reported that the intervention enhanced awareness of internal states (15.63%), was user-friendly (9.38%), and enjoyable (9.38%). The mentalization group more frequently reported increased mental state awareness compared to the mindfulness group (19.05% vs. 9.09%).
Common criticisms included interventions not being contextually relevant (15.63%), EMA prompts being too frequent (12.50%), confusing or irrelevant prompts (12.50%), and repetitive prompts (6.25%). The mentalization group more often reported intervention relevance issues than the mindfulness group (15% vs. 7.69%).
Suggestions for Improvement
Individual suggestions included modifying EMA response options (n= 2), adjusting prompt timing based on schedule changes (n=1), increasing compensation (n=1), and adding functionality to record supportive interactions (n=1)."

DISCUSSION

You're editing your response. Sharing this URL allows others to also edit your response.

FILL IN A NEW RESPONSE

22) Interpretation consistent with results, balancing benefits and harms, and considering other relevant evidence

NPT: In addition, take into account the choice of the comparator, lack of or partial blinding, and unequal expertise of care providers or centers in each group

22-i) Restate study questions and summarize the answers suggested by the data, starting with primary outcomes and process outcomes (use)

Restate study questions and summarize the answers suggested by the data, starting with primary outcomes and process outcomes (use).

	1	2	3	4	5	
subitem not at all important	○	○	○	◉	○	essential

Clear selection

Does your paper address subitem 22-i? *

Copy and paste relevant sections from the manuscript (include quotes in quotation marks "like this" to indicate direct quotes from your manuscript), or elaborate on this item by providing additional information not in the ms, or briefly explain why the item is not applicable/relevant for your study

"This pilot randomised controlled trial aimed to assess the feasibility, acceptability, and preliminary effects of mindfulness and mentalisation-based micro-interventions delivered via smartphone for individuals experiencing common mental health problems. Our results indicate that the intervention was feasible, with strong recruitment and retention rates, and high adherence to the EMI protocol. Participants reported high levels of acceptability, finding the interventions easy to use, helpful, and not burdensome. Preliminary effectiveness data revealed that both the mentalisation and mindfulness interventions were associated with significant within-group reductions in symptoms of anxiety and depression from pre- to post intervention, with a significant proportion of participants no longer meeting caseness criteria for GAD or depression post-intervention. There were no statistically significant differences between the mentalisation and mindfulness groups on post-intervention symptom scores. "

You're editing your response. Sharing this URL allows others to also edit your response.

FILL IN A NEW RESPONSE

22-ii) Highlight unanswered new questions, suggest future research

Highlight unanswered new questions, suggest future research.

	1	2	3	4	5	
subitem not at all important	○	○	○	◉	○	essential

Clear selection

Does your paper address subitem 22-ii?

Copy and paste relevant sections from the manuscript (include quotes in quotation marks "like this" to indicate direct quotes from your manuscript), or elaborate on this item by providing additional information not in the ms, or briefly explain why the item is not applicable/relevant for your study

"Further work is required to optimise the threshold for triggering the interventions. While EMIs share similarities with just-in-time adaptive interventions (JITAIs), JITAIs place a greater emphasis on the dynamic tailoring of interventions based on an individual's evolving context and needs (56). A significant hurdle lies in the selection and integration of existing empirical, theoretical, and practical evidence into a coherent scientific model that can effectively guide the design and development of these interventions (57). Identifying the specific factors that indicate a state of vulnerability or opportunity for intervention is a complex task, as the optimal timing for support can vary greatly depending on the individual and the context. Accurately assessing an individual's receptivity to an intervention at any given moment presents another challenge, as factors such as their current location, emotional state, and social environment can influence their willingness and ability to engage. " other examples throughout discussion

20) Trial limitations, addressing sources of potential bias, imprecision, and, if relevant, multiplicity of analyses

You're editing your response. Sharing this URL allows others to also edit your response.

FILL IN A NEW RESPONSE

20-i) Typical limitations in ehealth trials

Typical limitations in ehealth trials: Participants in ehealth trials are rarely blinded. Ehealth trials often look at a multiplicity of outcomes, increasing risk for a Type I error. Discuss biases due to non-use of the intervention/usability issues, biases through informed consent procedures, unexpected events.

	1	2	3	4	5	
subitem not at all important	○	○	○	◉	○	essential

Clear selection

You're editing your response. Sharing this URL allows others to also edit your response.

FILL IN A NEW RESPONSE

Does your paper address subitem 20-i? *

Copy and paste relevant sections from the manuscript (include quotes in quotation marks "like this" to indicate direct quotes from your manuscript), or elaborate on this item by providing additional information not in the ms, or briefly explain why the item is not applicable/relevant for your study

"These findings are subject to several important limitations. The small sample size (N=84 randomised) inherently limits the statistical power to detect significant differences, particularly when looking at differences across a number of measures. Preliminary efficacy findings, while showing promising within-group trends, should be interpreted with caution, while there were comparison arms, and each individual acted as their own control, for assessment of EMA outcomes, there was no passive control arm. An arm with EMA only would have been a useful addition, where EMA alone is likely to lead to greater self awareness (mentalisation of self) and bring about changes in mood (46,54).
This EMI approach employs a "less is more" philosophy, focusing on developing specific skills through repeated practice that transfers to daily life. However, implementation revealed significant variability in completion rates (0-100%) with no clear dose-response relationship, complicating effectiveness assessment. A notable challenge emerged in the disconnect between EMA assessment completion and actual engagement with the triggered micro-interventions. Participants often responded to assessment prompts while bypassing the therapeutic content. Qualitative feedback suggested several potential explanations: interventions sometimes lacked contextual relevance (particularly in the mentalisation group), prompts became overly frequent or repetitive, or participants struggled to engage with specific content at the moment of delivery. The simplicity of the strategies may also influence engagement patterns. Once learned, participants might implement strategies independently upon receiving prompts, without needing to follow the step-by-step guidance in the app each time. This self-directed application could explain intervention effectiveness despite lower formal completion rates. While EMA alone might produce some change effects, the observed differences between intervention groups on EMA items suggest specific intervention impacts beyond assessment effects. Post-intervention qualitative interviews would have provided valuable insights into these engagement patterns and helped distinguish between assessment and intervention effects. Future refinements should address these engagement challenges by modifying response options, adjusting prompt timing, and ensuring contextual relevance to maximize therapeutic engagement.
The short intervention duration (21 days of EMI) means the findings capture only immediate post-intervention changes and do not provide insight into the long-term sustainability of effects. Lack of long-term follow-up is a common limitation of pilot studies and restricts understanding of enduring clinical impact. Although retention was high overall, dropout did occur, and those who dropped out were significantly younger, suggesting potential differences in engagement based on age. The sample population, primarily recruited from a university setting and skewed towards younger adults, females, and those of Asian or Asian British, and White ethnicity, limits the generalisability of the findings to broader populations with common mental health problems (55)."

You're editing your response. Sharing this URL allows others to also edit your response.

FILL IN A NEW RESPONSE

21) Generalisability (external validity, applicability) of the trial findings

NPT: External validity of the trial findings according to the intervention, comparators, patients, and care providers or centers involved in the trial

21-i) Generalizability to other populations

Generalizability to other populations: In particular, discuss generalizability to a general Internet population, outside of a RCT setting, and general patient population, including applicability of the study results for other organizations

	1	2	3	4	5	
subitem not at all important	○	○	○	●	○	essential

Clear selection

Does your paper address subitem 21-i?

Copy and paste relevant sections from the manuscript (include quotes in quotation marks "like this" to indicate direct quotes from your manuscript), or elaborate on this item by providing additional information not in the ms, or briefly explain why the item is not applicable/relevant for your study

"Although retention was high overall, dropout did occur, and those who dropped out were significantly younger, suggesting potential differences in engagement based on age. The sample population, primarily recruited from a university setting and skewed towards younger adults, females, and those of Asian or Asian British, and White ethnicity, limits the generalisability of the findings to broader populations with common mental health problems (55)."

21-ii) Discuss if there were elements in the RCT that would be different in a routine application setting

Discuss if there were elements in the RCT that would be different in a routine application setting (e.g., prompts/reminders, more human involvement, training sessions or other co-interventions) and what impact the omission of these elements could have on use, adoption, or outcomes if the intervention is applied outside of a RCT setting.

1	2	3	4	5

You're editing your response. Sharing this URL allows others to also edit your response.

FILL IN A NEW RESPONSE

Does your paper address subitem 21-ii?

Copy and paste relevant sections from the manuscript (include quotes in quotation marks "like this" to indicate direct quotes from your manuscript), or elaborate on this item by providing additional information not in the ms, or briefly explain why the item is not applicable/relevant for your study

Your answer

OTHER INFORMATION

23) Registration number and name of trial registry

Does your paper address CONSORT subitem 23? *

Copy and paste relevant sections from the manuscript (include quotes in quotation marks "like this" to indicate direct quotes from your manuscript), or elaborate on this item by providing additional information not in the ms, or briefly explain why the item is not applicable/relevant for your study

https://aspredicted.org/kn7g-xkhg.pdf

24) Where the full trial protocol can be accessed, if available

Does your paper address CONSORT subitem 24? *

Cite a Multimedia Appendix, other reference, or copy and paste relevant sections from the manuscript (include quotes in quotation marks "like this" to indicate direct quotes from your manuscript), or elaborate on this item by providing additional information not in the ms, or briefly explain why the item is not applicable/relevant for your study

this was not applicable to the study

You're editing your response. Sharing this URL allows others to also edit your response.

FILL IN A NEW RESPONSE

Does your paper address CONSORT subitem 25? *

Copy and paste relevant sections from the manuscript (include quotes in quotation marks "like this" to indicate direct quotes from your manuscript), or elaborate on this item by providing additional information not in the ms, or briefly explain why the item is not applicable/relevant for your study

this is not applicable - no funding

X27) Conflicts of Interest (not a CONSORT item)

X27-i) State the relation of the study team towards the system being evaluated

In addition to the usual declaration of interests (financial or otherwise), also state the relation of the study team towards the system being evaluated, i.e., state if the authors/evaluators are distinct from or identical with the developers/sponsors of the intervention.

	1	2	3	4	5	
subitem not at all important	◉	○	○	○	○	essential

Clear selection

Does your paper address subitem X27-i?

Copy and paste relevant sections from the manuscript (include quotes in quotation marks "like this" to indicate direct quotes from your manuscript), or elaborate on this item by providing additional information not in the ms, or briefly explain why the item is not applicable/relevant for your study

The study team are evaluators who delivered and managed the intervention within the controlled trial environment using an existing app platform (mPath)

About the CONSORT EHEALTH checklist

You're editing your response. Sharing this URL allows others to also edit your response.

FILL IN A NEW RESPONSE

As a result of using this checklist, did you make changes in your manuscript? *

- ◯ yes, major changes
- ◉ yes, minor changes
- ◯ no

What were the most important changes you made as a result of using this checklist?

additional points to address the questions

How much time did you spend on going through the checklist INCLUDING making changes in your manuscript *

it took about two hours to complete

As a result of using this checklist, do you think your manuscript has improved? *

- ◯ yes
- ◉ no
- ◯ Other:

You're editing your response. Sharing this URL allows others to also edit your response.

FILL IN A NEW RESPONSE

Would you like to become involved in the CONSORT EHEALTH group?

This would involve for example becoming involved in participating in a workshop and writing an "Explanation and Elaboration" document

- ( ) yes
- (•) no
- ( ) Other:

Clear selection

Any other comments or questions on CONSORT EHEALTH

no we dont have any questions

STOP - Save this form as PDF before you click submit

To generate a record that you filled in this form, we recommend to generate a PDF of this page (on a Mac, simply select "print" and then select "print as PDF") before you submit it.

When you submit your (revised) paper to JMIR, please upload the PDF as supplementary file.

Don't worry if some text in the textboxes is cut off, as we still have the complete information in our database. Thank you!

Final step: Click submit !

Click submit so we have your answers in our database!

Submit

Never submit passwords through Google Forms.

This content is neither created nor endorsed by Google. - Contact form owner - Terms of Service - Privacy Policy

Does this form look suspicious? Report

Google Forms

You're editing your response. Sharing this URL allows others to also edit your response.

FILL IN A NEW RESPONSE

You're editing your response. Sharing this URL allows others to also edit your response.

FILL IN A NEW RESPONSE
